# Colored visual stimuli evoke spectrally tuned neuronal responses across the central nervous system of zebrafish larvae

**DOI:** 10.1186/s12915-020-00903-3

**Published:** 2020-11-27

**Authors:** Chiara Fornetto, Natascia Tiso, Francesco Saverio Pavone, Francesco Vanzi

**Affiliations:** 1 European Laboratory for Non-Linear Spectroscopy (LENS), via Nello Carrara 1, 50019 Sesto Fiorentino, FI Italy; 2 Department of Biology, University of Padova, via Ugo Bassi 58b, 35131 Padua, Italy; 3 Department of Physics and Astronomy, University of Florence, via G. Sansone 1, 50019 Sesto Fiorentino, FI Italy; 4 National Research Council (CNR), National Institute of Optics (INO), Largo Enrico Fermi 6, 50125 Florence, FI Italy; 5 Department of Biology, University of Florence, via Madonna del Piano 6, 50019 Sesto Fiorentino, FI Italy

**Keywords:** Color vision, Zebrafish, Calcium imaging

## Abstract

**Background:**

Visually guided behaviors such as optomotor and optokinetic responses, phototaxis, and prey capture are crucial for survival in zebrafish and become apparent after just a few days of development. Color vision, which in zebrafish is based on a spatially anisotropic tetrachromatic retina, provides an additional important component of world representation driving fundamental larval behaviors. However, little is known about the central nervous system (CNS) circuitry underlying color vision processing downstream of the retina, and its activity correlates with behavior. Here, we used the transparent larva of zebrafish to image CNS neurons and their activity in response to colored visual stimuli.

**Results:**

To investigate the processing of chromatic information in the zebrafish larva brain, we mapped with cellular resolution, spectrally responsive neurons in the larva encephalon and spinal cord. We employed the genetically encoded calcium indicator GCaMP6s and two-photon microscopy to image the neuronal activity while performing visual stimulation with spectrally distinct stimuli at wavelengths matching the absorption peaks of the four zebrafish cone types. We observed the presence of a high number of wavelength-selective neurons not only in the optic tectum, but also in all other regions of the CNS, demonstrating that the circuitry involved in processing spectral information and producing color-selective responses extends to the whole CNS.

**Conclusions:**

Our measurements provide a map of neurons involved in color-driven responses, revealing that spectral information spreads in all regions of the CNS. This suggests the underlying complexity of the circuits involved and opens the way to their detailed future investigation.

## Background

Most animal species depend strongly on their sense of vision; the ability to perceive and discriminate chromatic signals is very useful for object detection and identification in the natural environment [1]. Animals with a larval stage are generally characterized by a huge investment of energy in the rapid development of a complex retina [2], associated with downstream visual circuits, which improve the larva’s chances of survival with sensorial cues fundamental for both predator escape and prey capture. The larval zebrafish visual system is characterized by the presence of a spatially anisotropic retina with one rod photoreceptor type, becoming functional at around 15 days post-fertilization (dpf) [3], and four morphologically and spectrally distinct cone types (with opsins exhibiting absorption peaks at 362, 415, 480, and 570 nm [4]) distributed heterogeneously across the visual field [5]. Indeed, the environmental distribution of radiance, covering a wide range of wavelengths, may be used to construct a visual map guiding several behaviors (for example, prey capture), based on different world representations mapped with the different wavelengths [5–8]. Spectral information, based on such tetrachromatic retina, represents a fundamental sensory cue driving many larval behaviors.

Behavioral studies regarding optomotor response have shown a dependence on light wavelength, demonstrating that zebrafish larvae produce an optomotor response to red or green motion but not to the shorter wavelengths [9]. Phototaxis assays, on the other hand, have shown that larvae, when faced with a binary choice between two colors, display a clear chromatic preference, with an especially strong UV avoidance [10]. Thus, the wide visually guided behavioral repertoire [11–14], in combination with the optical transparency of the larva and the expression of genetically encoded calcium indicators, have made this organism a prominent vertebrate model for the study of visually evoked behaviors and of the neuronal circuits underlying them [15–19]. In order to investigate the processing of spectral information and the neuronal origin of color-driven behaviors, we applied two-photon calcium imaging to larvae at two different stages of development (3 and 5 dpf) undergoing visual stimulation with spectrally distinct stimuli. We identified and mapped, with cellular resolution in the whole larval brain, neurons with spectrally specific activity. Our results show that responsive neurons are concentrated with the highest density in the mesencephalon, but are also present in all other CNS regions investigated, including the spinal cord. These wavelength-selective neurons can propagate spectral information throughout the CNS and determine color-sensitive behaviors.

## Results

### Whole-brain imaging during visual stimulation

**Fig. 1.**
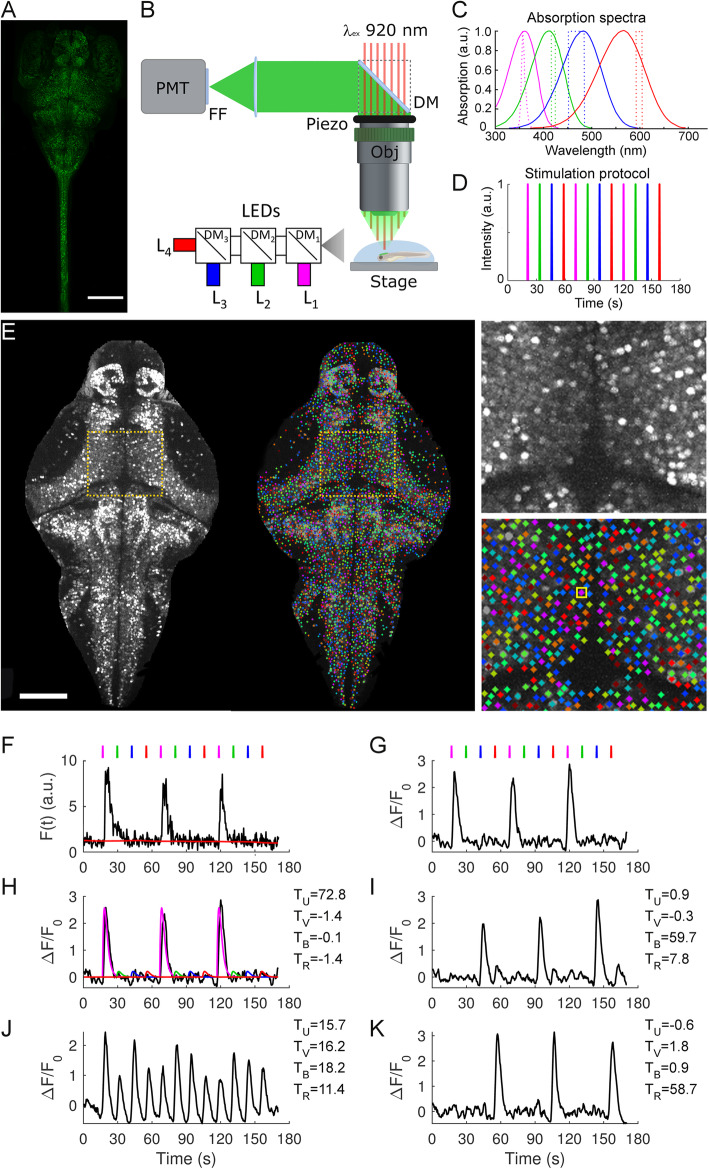
Experimental setup, visual stimulation protocol, and regression-based identification of responsive neurons. **a** H2B-GCaMP6s fluorescence maximum intensity projection of the CNS of a 5-dpf larva. Scale bar, 200 μm. **b** Schematic of two-photon microscope used for imaging zebrafish larva brain combined with visual stimulation. Stimuli are presented dorso-frontally using LEDs of four different wavelengths. GM, galvanometric mirror; FF, fluorescence filter; DM, dichroic mirrors. Obj, Olympus × 20, 0.95NA, water immersion; L_1_, L_2_, L_3_, and L_4_ are the LEDs used for visual stimulation. **c** Normalized absorption spectra of zebrafish cones (solid lines, modified from Guggiana-Nilo and Engert [10]) overlapped with the LEDs emission spectra (dashed lines) used for visual stimulation. **d** Protocol of visual stimulation. **e** GCaMP fluorescence of a selected plane of a 5-dpf larva viewed dorsally (left image) and corresponding segmentation image (right image). Each identified neuron is labeled with a distinct arbitrary color. The dotted orange squares show the area magnified in the right panels, displaying cellular resolution of imaging (top) and segmented neurons (bottom). The inset in the bottom panel shows a neuron selected for the examples in **f**–**h**. Scale bar, 100 μm. **f** Fluorescence intensity *F*(*t*) measured in the 13-pixel cluster attributed to the selected cell highlighted in **e** (*F*_0_ is shown with the red line; see the “Methods” section for the detailed description of *F*_0_ and Δ*F*/*F*_0_ calculation). Stimulus time points are shown at the top. **g** Δ*F*/*F*_0_ trace. **h** Fitted regression data (colored trace) and corresponding *T* values. **i**–**k** Examples of Δ*F*/*F*_0_ traces and calculated *T* values obtained from the regression analysis on other neurons demonstrating the responsiveness to the different stimuli

**Fig. 2.**
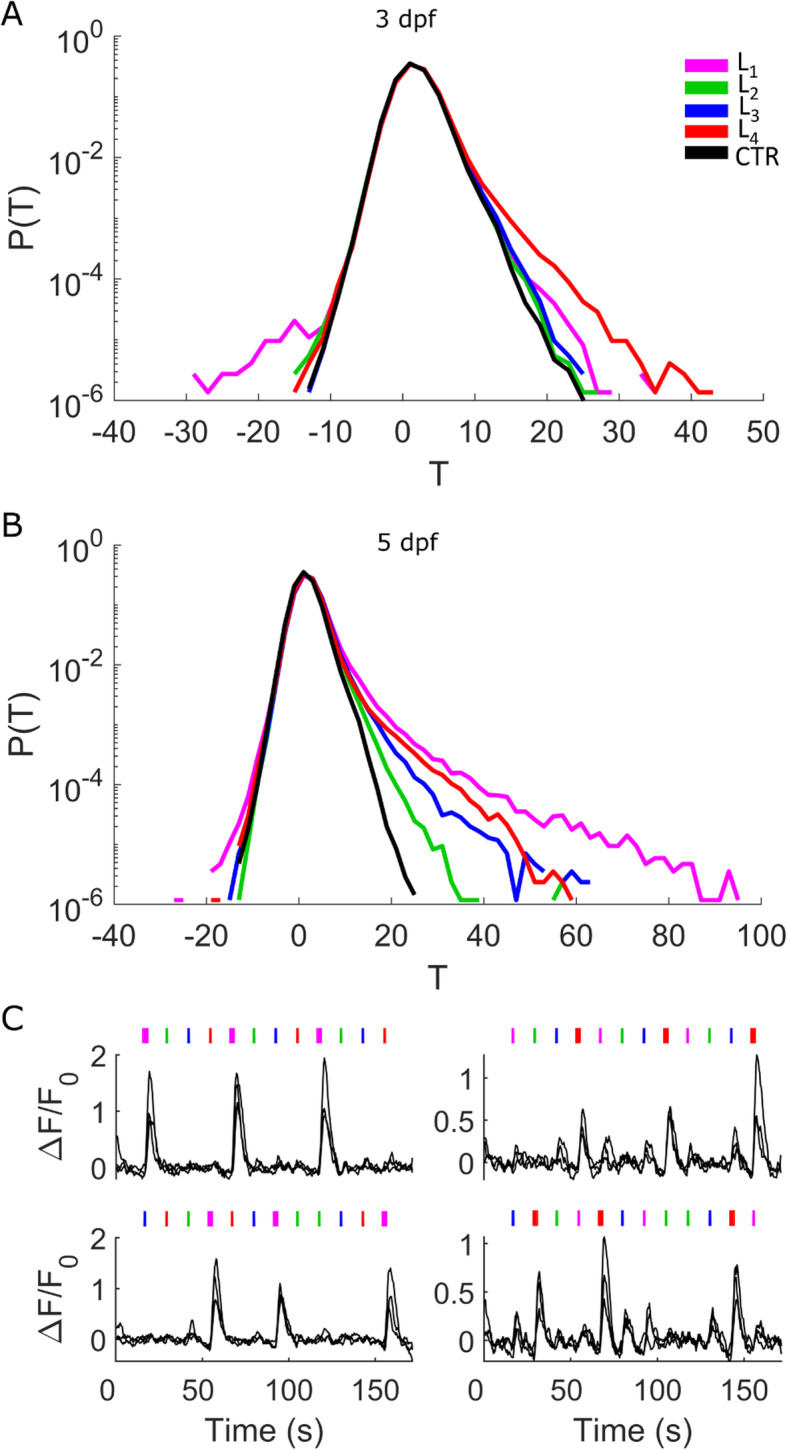
*T* distributions for stimulated and control larvae at 3 and 5 dpf and spectral identity. Normalized distributions of *T* values for L_1_, L_2_, L_3_, and L_4_ stimulation (magenta, green, blue, and red traces, respectively; see legend) and control acquired in the absence of visual stimulation (black trace) measured for 3-dpf larvae (**a**) and 5-dpf larvae (**b**). *N* = 7 larvae for stimulated data; *N* = 5 larvae for control data in both panels; it should be noted that statistics reported in the figure are cumulated over the total number of ROIs segmented over all larvae for each condition: 847,068 (3 dpf stimulated); 588,198 (3 dpf control); 845,070 (5 dpf stimulated); 518,409 (5 dpf control). **c** Example traces of neurons with spectrally selective responses under standard (top panels) and shuffled (bottom panels) protocol of visual stimulation. The left panels show Δ*F*/*F*_0_ traces of three neurons with a selective L_1_ response; the right ones show three neurons with selective L_4_ responses. Stimulus time points are shown at the top (thicker lines highlight the stimulus eliciting the selective response)

Two-photon microscopy was used to image the whole brain and part of the spinal cord of Tg (elavl3:H2B-GCaMP6s) larvae expressing at pan-neuronal level the genetically encoded calcium indicator GCaMP6s (Fig. 1a). The microscope was equipped with four LEDs (Fig. 1b) for visual stimulation at wavelengths matching the absorption peaks of the four zebrafish retina cone types (Fig. 1c). The direction of stimulus presentation corresponded to the stimulation of mainly the dorsal-frontal area of the visual field. Although we narrowed the emission bandwidth of each stimulus to less than 35 nm, the cone absorption spectra themselves are broad enough to determine an amount of insuppressible cross-talk, with the only exception of L cones for *λ* > 590 nm. We estimated for each LED the following relative excitation coefficients: L_1_ 1/0.54/0.04/0 for UV/S/M/L cones, respectively; L_2_ 0/1/0.37/0; L_3_ 0/0.14/1/0.22; and L_4_ 0/0/0/1. Throughout the paper, for simplicity and adopting a terminology related to human perception, we will sometimes refer to L_1_, L_2_, L_3_, and L_4_ stimuli also as UV, violet, blue, and red, representing the corresponding data in the figures with magenta, green, blue, and red, respectively. We recorded neuronal activity in 3- and 5-dpf larvae during visual stimulation with 100-ms light flashes characterized by the spectra shown in Fig. 1c and presented in the order L_1_, L_2_, L_3_, and L_4_ in triplicate (Fig. 1d). Three-dimensional mosaic two-photon fluorescence acquisition covered a volume of 1500 × 720 × 270 μm^3^ encompassing the whole encephalon and about one quarter of the spinal cord of the larva. Cellular resolution of imaging and automatic segmentation (Fig. 1e) allowed us to extract GCaMP6s fluorescence and Δ*F*/*F*_0_ traces for each segmented neuron (Fig. 1f, g). We identified about 100,000 (5 dpf) and 90,000 (3 dpf) neurons in the encephalon and about 10,000 (5 dpf) or 3700 (3 dpf) in the part of the spinal cord investigated (consisting of 8 segments). We applied linear regression analysis on the Δ*F*/*F*_0_ traces to measure each cell’s *T*-score for each of the four stimuli (Fig. 1h–k, see also the “Methods” section). Figure 2 shows the distributions of the four *T*-scores for 3- and 5-dpf larvae (*N* = 7 each), compared to controls recorded in the absence of stimuli (*N* = 5 each). The deviation of the *T* distributions obtained from stimulated larvae from those of control larvae highlights populations of neurons responding to the visual stimuli. At 3 dpf (Fig. 2a), we identify a larger fraction of neurons responding to L_4_ stimuli, followed by L_1_ and, less numerous, L_3_ and L_2_ stimuli. The *T* distribution at 5 dpf (Fig. 2b), on the other hand, displays noticeable differences with respect to 3 dpf, both quantitatively (a much larger proportion of spectrally responsive neurons can be observed with many of them characterized by higher *T*-scores than those measured at 3 dpf) and qualitatively (L_1_-responsive neurons are predominant, followed, in descending order, by L_4_, L_3_, and L_2_). In addition to neurons with positive *T* values above the control distribution, a small population is also detected with negative *T* values, which indicate a negative response to the light flashes, i.e., an inhibitory effect of light on neuronal activity. The differences among the four experimental distributions point at an appreciable degree of spectral identity for the populations of neurons involved (examples of unique vs. generalized spectral responses can also be seen in Fig. 1h–k). Before proceeding to a detailed spectral characterization of responsive neurons, we assessed the robustness of our experimental protocol and data analysis method. Regarding analysis, the results shown in Additional file 1: Fig. S1 (*T* distributions obtained by analyzing the data with a shuffled order of regressors) demonstrate the spectral specificity of *T* distributions shown in Fig. 2a, b. Regarding the stimulation protocol, we evaluated possible history-dependence effects by performing stimulation of the same neuron populations across the larva brain volume with two different stimulation patterns: our standard stimulus pattern L_1_, L_2_, L_3_, and L_4_ presented in triplicate and a shuffled pattern L_3_, L_4_, L_2_, L_1_, L_4_, L_3_, L_1_, L_2_, L_2_, L_3_, L_4_, and L_1_. Figure 2c shows example traces of the same neurons under the two different stimulation protocols, demonstrating maintenance of their spectral identity independently of the order of stimuli presentation (more on this in the “Discussion” section).

### Spectral identity and anatomical maps of responsive neurons

**Fig. 3.**
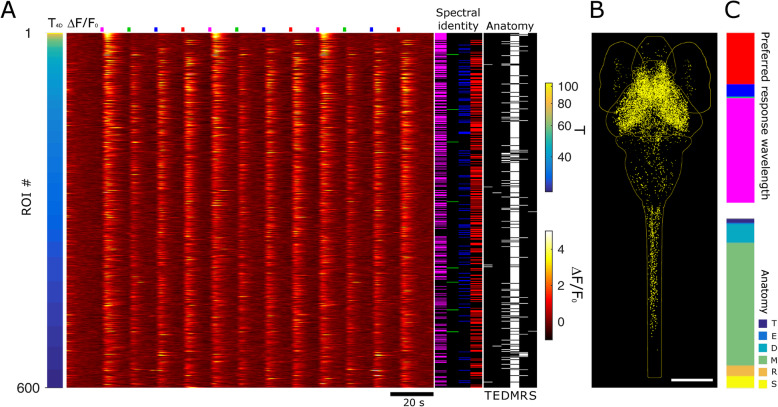
Stimulus-induced neuronal activity: spectral and anatomical mapping. **a** Heatmap of 600 ROIs ranked by *T*_4D_ values in a 5-dpf larva. The bar on the left shows *T*_4D_ values (mapped with the *T* color scale shown on the right of the panel). The main panel shows Δ*F*/*F*_0_ calcium responses of the 600 neurons (mapped with the Δ*F*/*F*_0_ color scale shown on the right of the panel). Stimulus time points are indicated in their respective colors above the panel. The spectral identity column (next to the heatmap) indicates the spectral identity of each neuron as determined by the threshold analysis: magenta, green, blue, and red lines indicate neurons responsive to L_1_, L_2_, L_3_, and L_4_ stimuli, respectively; black indicates no response to the visual stimulus. The anatomy column shows the localization of each of the 600 neurons (T, telencephalon; E, eyes; D, diencephalon; M, mesencephalon; R, rhombencephalon; S, spinal cord). Scale bar, 190 μm. **b** Anatomical map of neurons selected above the *T*_4D_ threshold (yellow dots) for 7 larvae at 5 dpf registered onto a reference brain (outlines of the anatomical regions mentioned above are displayed in light yellow). **c** Quantification of spectral and anatomical identities (*N* = 7 larvae). The top bar graph shows the normalized distribution of all responsive neurons above the *T*_4D_ threshold across the four spectral stimuli (with their respective color code). The bottom bar graph quantifies the anatomical distribution of the neurons across the CNS areas with the following color coding from top to bottom: dark blue, telencephalon; light blue, eye; turquoise, diencephalon; green, mesencephalon; orange, rhombencephalon; and yellow, spinal cord. The same data with standard errors are shown in Additional file 3:Fig. S3

**Fig. 4.**
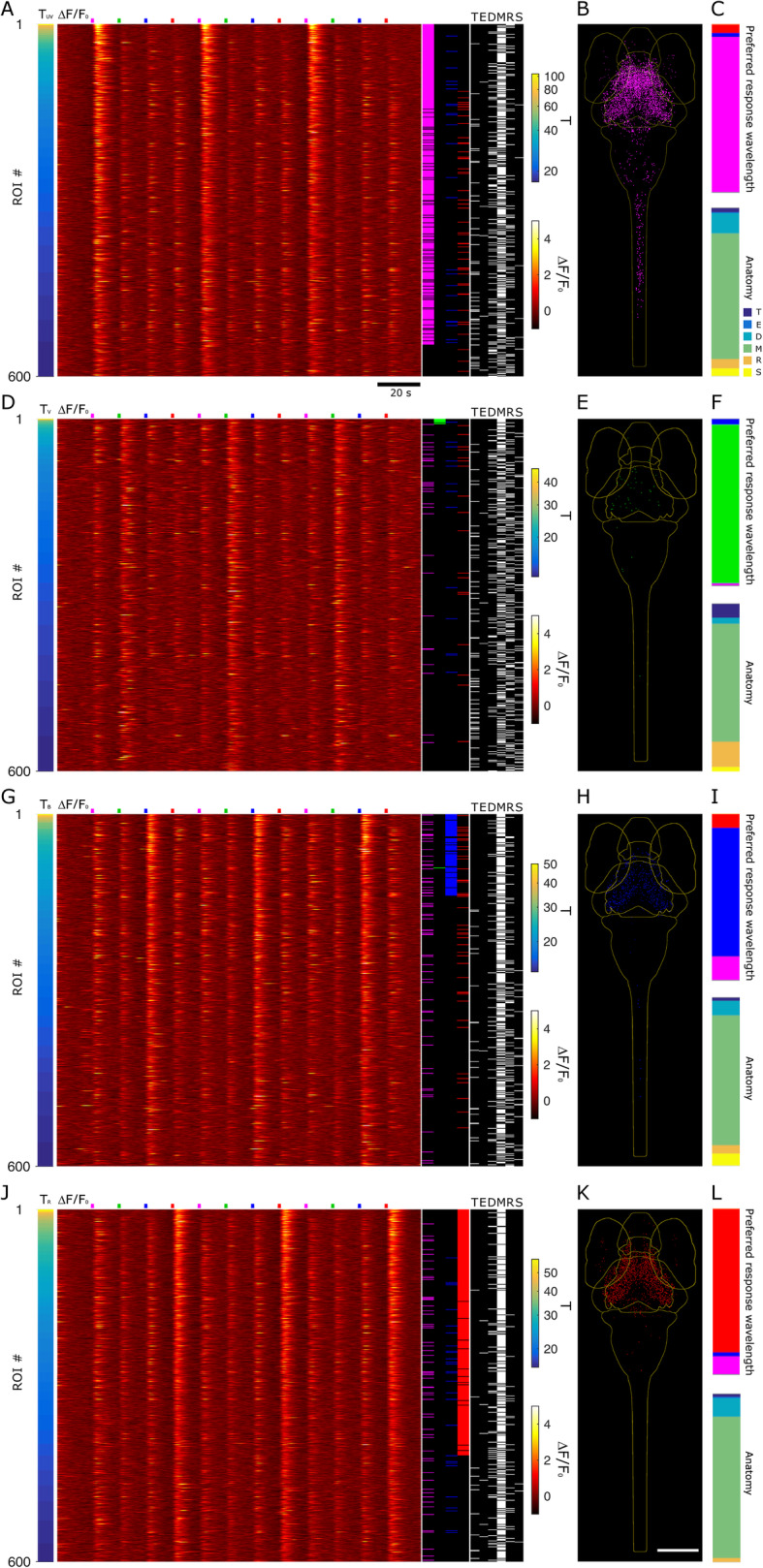
Spectrally tuned responses and anatomical distributions. Δ*F*/*F*_0_ heatmaps, spectral identity, and anatomical distributions of neurons selected for spectrally tuned responses above the threshold for UV (**a**–**c**), violet (**d**–**f**), blue (**g**–**i**), and red (**j**–**l**) stimuli. See Fig. 3 for the general panel description. **a**, **d**, **g**, **j** Data from the same 5-dpf larva used for Fig. 3 but with neurons ranked based on L_1_, L_2_, L_3_, and L_4_ responses, respectively, as shown by the leftmost column. Data from all seven 5 dpf larvae, displaying neurons that passed selection criteria (threshold and peak analysis) for on L_1_ (**b**, **c**), L_2_ (**e**, **f**), L_3_ (**h**, **i**), and L_4_ (**k**, **l**) responses, respectively. Anatomical distributions of all responsive neurons (**b**, **e**, **h**, **k**), preferred response wavelength, and anatomy are shown (**c**, **f**, **i**, **l**). Scale bar, 190 μm

**Fig. 5.**
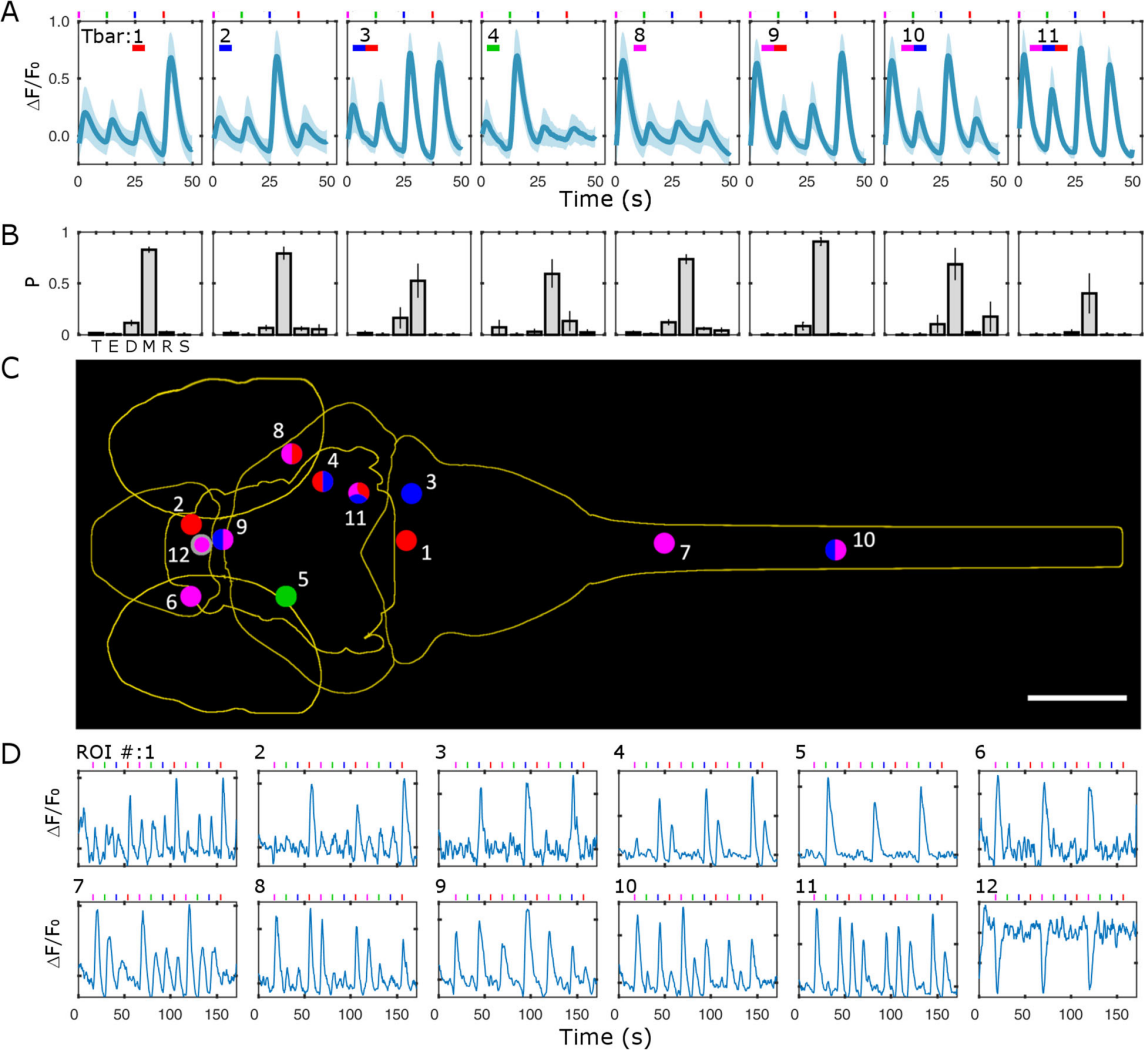
Unique spectral classification with Tbar. **a** Average Δ*F*/*F*_0_ traces (blue line, mean; light blue area, standard deviation) for all neurons identified in seven 5-dpf larvae with the given Tbar (indicated in each panel). Number of neurons: 2243 (Tbar 1), 581 (Tbar 2), 49 (Tbar 3), 51 (Tbar 4), 4509 (Tbar 8), 308 (Tbar 9), 88 (Tbar 10), and 14 (Tbar 11). The experimental trace of each neuron was first averaged over the three repetitions of the L_1_/L_2_/L_3_/L_4_ stimuli before averaging among different neurons. Standard deviations were calculated over all data (made of the three repetitions for each neuron, over all neurons found in each class for the seven larvae tested). Colored tick marks above each graph show the timing of the different stimuli. Only the classes with a total numerical consistency above 10 neurons are shown in the figure. **b** Anatomical distributions of neurons for each Tbar class. Anatomical areas are classified and indicated as in the rest of the paper: Telencephalon (T), eye (E), diencephalon (D), mesencephalon (M), rhombencephalon (R), and spinal cord (S). Values shown are averages and stderr calculated over the seven normalized distributions measured with the 7 larvae for each Tbar. The bars for the last plot (Tbar 11) do not add to 1 because the distributions were normalized to 1 for each larva across the anatomical areas, but not all larvae tested actually contributed cells to this Tbar class (i.e., some larvae contributed 0 into averaging). **c** Anatomical localization of some example neurons for each of the Tbar classes shown in the previous panels. Each neuron is shown as a dot (not drawn to scale, for visibility) and colored according to its responses with the color coding used throughout the paper. The gray border around neuron #12 indicates a negative *T* value. Scale bar, 150 μm. **d** Experimental Δ*F*/*F*_0_ traces for the neurons shown in **c**. The scales were chosen to optimize visibility in each graph and are not shown for compactness of the figure; the two ticks shown on the vertical axis are as follows for each panel starting from top left: (− 0.5, 2.2), (− 0.3, 1.2), (− 0.3, 1.4), (− 0.6, 4.3), (− 0.5, 3.7), (− 0.3, 1.4), (− 0.6, 2.1), (− 0.5, 3.0), (− 0.3, 1.3), (− 0.4, 3.4), (− 0.5, 4.0), and (− 0.4, 0.2)

**Fig. 6.**
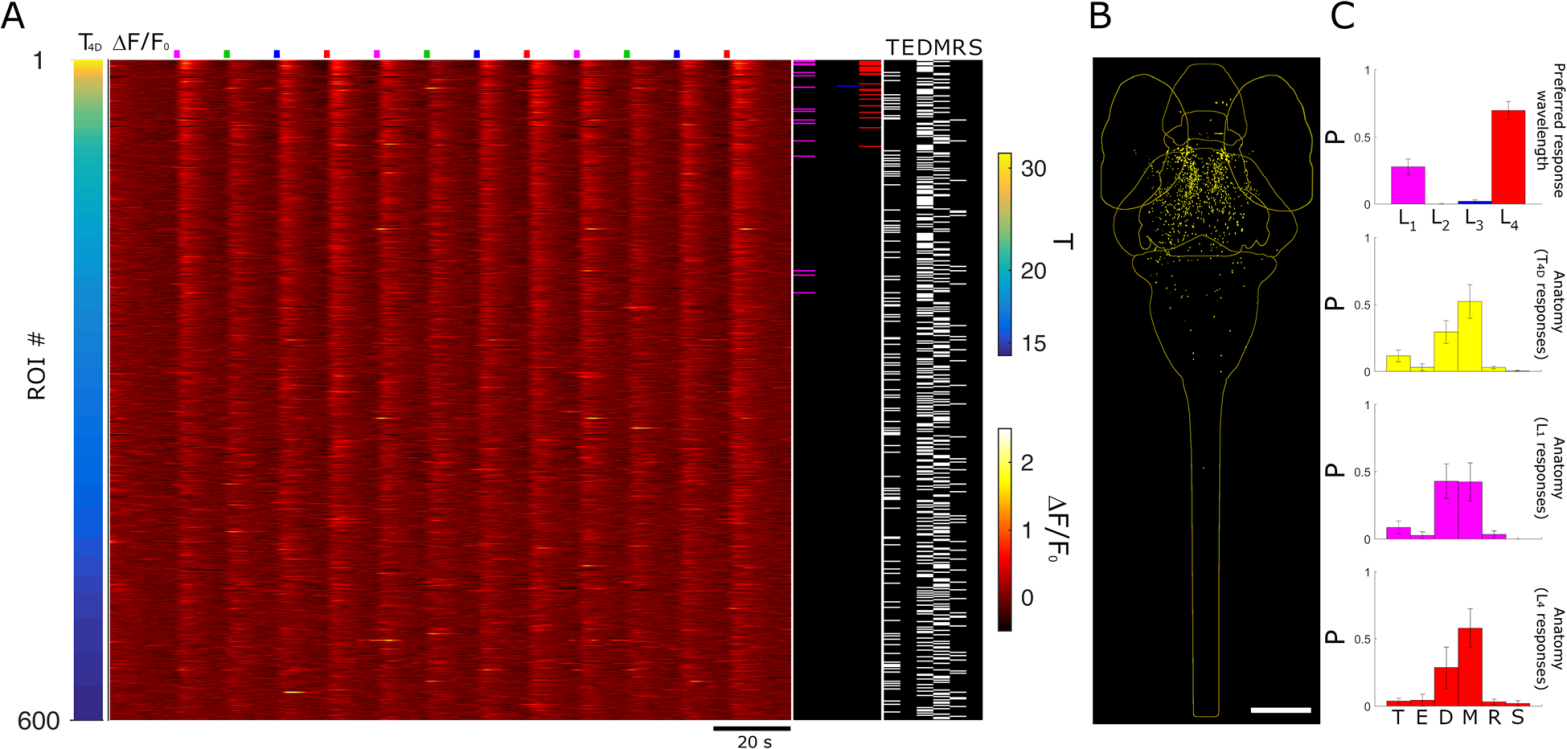
CNS spectral and anatomical mapping in 3-dpf larvae. **a** Δ*F*/*F*_0_ responses of 600 ROIs ranked by *T*_4D_ values in a 3-dpf larva. Each row in the heatmap represents a neuron. The left bar indicates neurons’ *T*_4D_ values (*T* color scale is displayed on the right of the panel). Stimulus time points are indicated at the top of the panel highlighted in their respective colors. The spectral column on the right shows the spectral responses of the neurons selected above the threshold and peak analysis (highlighted in magenta, green, blue, and red for the response to L_1_, L_2_, L_3_, and L_4_ stimulus, respectively). The anatomical column shows the localization of each ROI across the CNS regions (T, telencephalon; E, eyes; D, diencephalon; M, mesencephalon; R, rhombencephalon; S, spinal cord). **b** Anatomical map of neurons selected above the *T*_4D_ threshold (yellow dots) for 7 larvae at 3 dpf registered onto a reference brain for anatomical localization (light yellow outlines). Scale bar, 150 μm. **c** Quantification of spectral and anatomical identities (*N* = 7 larvae). The histogram on the top shows the normalized distribution of all responsive neurons above the *T*_Th4D_ threshold across the four spectral stimuli (displayed in their respective color codes). The histograms on the bottom quantify the anatomical distributions of the neurons above *T*_Th4D_ (yellow histogram), *T*_ThU_ (magenta), and *T*_ThR_ threshold (red) (from top to bottom) across the CNS areas abovementioned. Error bars = stderr, *N* = 7

To spectrally characterize the populations of responsive neurons, we proceeded with the identification of cells reliably responding to the visual stimuli. We first aimed at identifying all neurons responding to any of the stimuli in our measurements. Thus, we defined ${T}_{4D}=\sqrt{T_U^2+{T}_V^2+{T}_B^2+{T}_R^2}$ which quantifies neuron activity triggered by the stimuli but without spectral selection. Figure 3a shows data ranked by *T*_4D_ for 600 neurons in one 5-dpf larva. The Δ*F*/*F*_0_ heatmap shows the strong calcium responses associated with the visual stimuli. We quantified each neurons’ spectral response based on its four *T* values associated with each stimulus wavelength. The selection of neurons significantly responding to the stimuli is based on thresholds determined by quantitative comparison of experimental and control *T* distributions and calculation of false discovery rates (FDR) as described in the “Methods” section. Additional file 2: Fig.S2 shows the plots of FDR vs. *T* threshold (*T*_Th_); the data lead to the selection of *T*_Th4D_ = 18.5, *T*_ThU_ = 17, *T*_ThV_ = 24.7, *T*_ThB_ = 20.8, and *T*_ThR_ = 18.1. The right columns of Fig. 3a show the spectral identity and anatomical localization of the 600 neurons. Inspection of these data shows that high *T*_4D_ values are associated especially with L_1_ and L_4_ stimuli and distributed mainly, but not exclusively, in the mesencephalon. Figure 3b shows all neurons above *T*_Th4D_ for 7 larvae registered onto a reference brain (see the “Methods” section; ref. [20]). The density of neurons observable in the different regions confirms the trend highlighted for one larva in Fig. 3a. Spectral and anatomical identities of all neurons in the seven larvae are quantified in Fig. 3c and Additional file 3: Fig.S3. As anticipated by the distribution of Fig. 2b, the highest fraction of neurons is responsive to L_1_, followed by L_4_, L_3_, and a very small number to L_2_. The anatomical data, on the other hand, show the highest density in the mesencephalon. The obseravtion that the density of spectrally responsive neurons is highest in this regione of the CNS is expected, since it comprises the principal areas for processing visual (along with other sensorial) information (for example, the optic tectum and the other retino-recipient areas). However, we also identified positive neurons in all other encephalic areas and in the spinal cord, indicating that the circuitry in charge of processing spectral information and color-selective responses is not limited to the mesencephalon. The ranking based on *T*_4D_ provides an overall view of spectral responses; to assess each stimulus specifically, we ranked the neurons based on the four respective *T*s (Fig. 4a, d, g, j). The Δ*F*/*F*_0_ signals shown in the heatmaps demonstrate the dominance of the stimulus selected for ranking over the others in each panel, with L_1_ and L_2_ generally providing the stronger and the weaker responses, respectively. Note that spectral ranking provides a distribution of neurons based on a single *T*, thus showing cells responding (with 99% confidence) to that stimulus, independently on the possibility that a neuron may respond to multiple stimuli (more on this below). The anatomical panels for the selected larva (Fig. 4a, d, g, j, right columns) and cumulative anatomical distributions for the seven larvae (Fig. 4b, e, h, k) confirm the expected dominance of mesencephalic localization and reveal a density of neurons which is highest for those responsive to L_1_, followed by L_4_ and L_3_, and a very small number of L_2_. The anatomical distributions shown in Fig. 4c, f, i, l highlight that neurons in the spinal cord participate in responses only to the shorter wavelengths, lacking response to the L_4_ stimulus. The spectral panels (Fig. 4a, d, g, j) demonstrate the existence of some neurons responding to multiple wavelengths. Due to the sequential stimulation in our experiments, we can assess the responsivity of each neuron to any of the stimuli and uniquely identify it based on the stimulus or stimuli it is sensitive to. We define a classifier “*Tbar*” (see the “Methods” section) which takes values from 1 to 15 depending on the stimulus response of the neuron. Figure 5a shows the average Δ*F*/*F*_0_ traces for all neurons belonging to *Tbar* classes significantly populated (*n* > 10 neurons) for the seven larvae of this work. The traces demonstrate the complexity of responses that are elicited in the larva CNS by the visual stimuli. *Tbar* classes 1, 2, 4, and 8 classify neurons with single wavelength selectivity for red, blue, violet, and UV stimuli, respectively; *Tbar* classes with responses to multiple wavelengths shown in the figure are classes 3 (red and blue response), 9 (red and UV), 10 (UV and blue), and 11 (UV, blue and red). The numerical consistency of each *Tbar* class reflects the spectral responses shown in Fig. 3, while the anatomical distributions (Fig. 5b) highlight again that longer wavelengths (L_4_) are not represented in the caudal regions (i.e., rhombencephalon and spinal cord) where, on the other hand, shorter wavelengths (L_1_, L_2_, L_3_) are represented singly or in combination. Figure 5c, d shows the anatomical localization (Fig. 5c) and corresponding Δ*F*/*F*_0_ traces (Fig. 5d) for some examples of neurons belonging to the *Tbar* classes in Fig. 5a. The experimental traces clearly demonstrate the stimulus-induced and spectrally selective nature of the response, confirming the robustness of the selection criteria adopted. As observed in *T* distributions shown in Fig. 2, some neurons exhibit a negative response to light (mainly UV). The last panel of Fig. 5d shows the Δ*F*/*F*_0_ trace of one of these neurons belonging to the pineal region (neuron #12 in Fig. 5c). Figure 6 shows the results obtained in 3-dpf larvae. Figure 6a displays the Δ*F*/*F*_0_ responses measured in one larva and ranked based on *T*_4D_. Comparison with Fig. 3a highlights the much weaker calcium responses measured at 3 dpf. The spectral column of the figure also shows a much smaller fraction of responsive cells, with an apparent shift in favor of L_4_. The anatomical column, on the other hand, shows a more evenly scattered distribution of the higher ranked neurons across the encephalic regions. Figure 6b shows all responsive neurons identified in 7 larvae after registration onto a reference brain. The histogram of spectral responses (Fig. 6c, top panel) indicates a strong dominance of L_4_ responses followed by L_1_ and a negligible response to L_2_ and L_3_. We then quantify the anatomical localization of neurons (Fig. 6c, lower panels) from these data. We can see that the mesencephalic dominance is not yet fully established at this stage of development. Moreover, the response to multiple wavelengths (which might be, to some extent, an indication of integration activity) is extremely rare (only the *Tbar* classes 1 and 8 are populated with, respectively, 423 and 122 neurons across seven larvae). We can notice that at 3 dpf, we found no neurons with spectral specificity in the spinal cord, indicating that, at this stage of development, the circuitries carrying spectrally dependent information down to the spinal cord and, thus, possibly eliciting “color”-selective motor behaviors are not yet functional. In opposition to the scarcity of positively responsive neurons (compared to 5 dpf), we consistently find the negatively responsive neurons also at 3 dpf. Additional file 4: Fig. S4 shows the anatomical localization and an example trace for these neurons which belong to the pineal region. Thus, we conclude that, while visual circuits are not yet fully developed, the inhibitory effect of light in the pineal gland is here demonstrated at the single-cell level already at 3 dpf.

## Discussion

In this work, we map, with cellular resolution, spectrally selective responses in the whole brain and part of the spinal cord of 3- and 5-dpf zebrafish transgenic larvae expressing the nuclear-localized calcium indicator GCaMP6s. We implement mosaic two-photon fluorescence imaging covering a volume of 1500 × 720 × 270 μm^3^ encompassing the whole encephalon and about one quarter of the spinal cord of the larva and measure neuronal activity during visual stimulation with light flashes with wavelengths centered on the absorption peaks of the four zebrafish retinal cones [4, 21, 22]. The use of IR light in two-photon microscopy, although not totally suppressing it, reduces the problem of direct retinal photoreceptors excitation [23], enabling imaging of neuronal activity with limited interference with our protocol of visual stimulation. Spectrally distinct stimuli elicit a strong and reproducible response in a subset of neurons of the larva CNS. We quantify such responses by linear regression and *T* score analysis. Observation of the distributions in Fig. 2 shows that control data fall to zero at *T* ~ 27, whereas stimulated larvae display a fraction of neurons characterized by *T* > 27. Identification of responsive neurons requires a choice of *T* threshold (*T*_Th_): imposing a value of *T*_Th_ as high as 27 would ensure a zero false-positive rate (FDR), but at the same time, it would miss a significant fraction of actually responsive neurons (giving rise to false negatives), since deviation of the experimental distributions from control is evident in Fig. 2 starting at *T* ~ 10. Thus, for 10 < *T*_Th_ < 27, a compromise has to be reached between false positives and false negatives. We chose to set the FDR at 1%, ensuring that the subsequent analysis selects positive neurons with 99% confidence. Indeed, the Δ*F*/*F*_0_ traces of the neurons selected with this criterion correlate strongly with the light flashes used in our experiments (as shown by the heatmaps in Figs. 3 and 4). Spectral characterization of neurons leads to the identification of single and multiple wavelength responses (see, for example, Figs. 3 and 4). We characterized this aspect with the unique identifier *Tbar* and showed the most relevant classes of neurons in Fig. 5. Even considering the insuppressible cross-talk between some of our excitation sources and the broad cone absorption spectra, we notice that the most populated *Tbar* classes clustering neurons with responses to multiple wavelengths cannot be explained based on cross-talk. For example, neurons responding to both L_1_ and L_4_ must receive independent inputs from these two channels since there is no cross-talk between these two sources (see Fig. 1c). We assessed the robustness of the *Tbar* analysis by evaluating the emergence of the classes shown in Fig. 5 in a spectrally unbiased analysis. The data shown in Additional file 5: Fig. S5 demonstrate that single-color classes are strongly populated across *T*_4D_ values and that these, as well as the multiple wavelength classes, emerge roughly with the same proportion even when lowering all the thresholds applied to a single value. We notice here that threshold analysis intrinsically imposes a compromise between false positives and false negatives; our choice in this regard was very stringent, unavoidably leading to some false negatives (as exemplified by the trace shown in Fig. 1j), but at the same time ensuring the reliable selection of neurons with very strong responses across the CNS. Nevertheless, the involvement of neurons in non-visual areas requires an assessment of potential artifacts due to spontaneous motor activity. In addition to 1% FDR selection based on the *T* threshold, therefore, we implemented a peak analysis to filter out the neurons not responding consistently to each of the three repetitions of the stimulus (see the “Methods” section). Example experimental traces (Figs. 1 and 5d), heatmaps (Figs. 3 and 4), and averaged traces (Fig. 5a) demonstrate the robustness of the neuron selection methods applied. We also tested possible history-dependence effects of the stimulation protocol. Additional file 6: Fig. S6 shows the correlation analysis and *Tbar* clustering obtained comparing the responses of the same neurons to different orders of the stimuli, demonstrating that the majority of neurons do not change their spectral identity with the order of the stimuli. We also characterized the fluorescence time response of all selected neurons, and a histogram of peak time points is shown in Additional file 7: Fig. S7. The peak distributions confirm the stimulus-triggered calcium dynamics based on the kinetics of GCaMP6s in our system. We should note that our choice of regressors unavoidably leads to the selection of those neurons exhibiting this type of response while, for example, slower response neurons would be missed. Also, no discrimination between ON and OFF responses can be applied to our data, since the duration of our stimulus is much shorter than the response of the GCaMP6s itself. Based on the importance of visual cues already in the larval stages and the consequent evolutionary investment in the processing of visual information, the numbers of positive cells we identified (representing about 2% of the total number of neurons in the CNS) might seem surprisingly low. However, it is important to consider the nature of the stimulus used in our experiments. Uniform illumination of the retina with monochromatic light would indeed produce a large amount of suppression in the retinal output, due to integration processes (for example, lateral inhibition) occurring in the retina itself [2, 24–26]. Our stimulus, on the other hand, can be assimilated to the perception of uniform illumination such as in phototaxis and chromatic preference experiments [10] and also in uniformly illuminated environments in general [27–31]. We should also note that, as discussed above, our threshold analysis has given priority to minimize FDR, unavoidably accepting a significant proportion of false negatives. Therefore, the percentage estimated is certainly an underestimate of the total number of neurons involved in response to our stimuli. Another issue related to intra-retinal processing and contributing to reducing the number of stimulated neurons via signal suppression regards the intensity of the stimuli. In this work, we aimed at performing our measurements in a condition of saturation. Thus, we tested three intensities (0.1, 0.5, 1 mW/cm^2^) for each color and compared the *T* distributions obtained at each power with the control distribution. Our data (Additional file 8: Fig. S8) show that all responses but the one to L_4_ exhibit saturation already at 0.1 mW/cm^2^, while L_4_ reaches saturation at 0.5–1 mW/cm^2^. We, therefore, decided to set stimulus intensity at 1 mW/cm^2^ for all LEDs. Undoubtedly, the dependence of response distributions on intensity, ON/OFF responses, color opponency, patterns, dynamics, and direction of stimulus presentation is extremely interesting areas of future investigation. Also, a full understanding of the whole process of visual input and signal processing leading to visually guided behaviors requires mapping activity in the whole CNS. Several works have been published dealing with either the retinal [5, 6, 29, 32–34] or the encephalic [18, 30, 35–38] side of this story. In a recent preprint from the Engert Lab [39], a double-labeling technique was applied to measure activity in both retinal ganglion cells (RGCs) and retinorecipient areas (RAs) of the encephalon. Our work extended measurements to the whole encephalon showing the extent of spectrally selective responses in all its regions. The anatomical maps shown in Figs. 3 and 4 highlight a high density of neurons responsive to L_1_, followed by L_4_ and L_3_ radiation, while only a very small subset of neurons responding to L_2_ stimuli. There are several reasons concurring to explain this observation. Firstly, we need to consider the high anisotropy of the larva visual system [5]. The position of our stimulation LEDs causes illumination of the dorsal-frontal region of the visual field, stimulating the area temporalis (also termed “strike zone”) which is particularly rich in UV cones and UV-on circuits [5, 32] and is implied in prey capture [6]. In addition to UV, Zhou et al. [32] highlighted in this area strong responses also to red light, thus anticipating that the projections from the strike zone to the brain would mostly involve these wavelengths, as we indeed observed. Secondly, it was demonstrated that UV cones develop first and are the most abundant in the larval retina, followed by blue and by the other cone types [8, 40–43]. In addition to the direction of stimulation, we need to consider the nature of our stimulus: we performed brief (100 ms) flashes with narrow spectral band matching, as closely as possible, the four zebrafish cone types [4, 21, 22]. Uniform illumination and insuppressible spectral cross-talk (occurring in all cases but L_4_, see Fig. 1c) elicit a high degree of processing within the zebrafish retina itself [41, 43, 44] before reaching the RGCs. RGCs are, in turn, the source of signal for the downstream encephalic neurons, which are the object of our measurements. Thus, rather than mapping the responses due to the activity of each cone type individually, we map neurons involved in the perception and processing of information which we may link to specific “colors” as perceived by the larva for the four spectra used in our stimulation. The pathway of visual information processing, starting from photoreceptors up to the RGCs, becomes functional at 70–74 hpf and is necessary in establishing contacts with the optic tectum (OT, 41). Connaughton and Nelson [45] demonstrated the capability of larval RGCs to spectrally discriminate light of different wavelengths, showing a prominent response to UV light. This capability was evident in zebrafish larvae starting from 5 dpf, explaining the strong UV response we observe at 5-dpf compared to 3-dpf larvae. A recent preprint from Engert Lab [39] discusses the projection of spectral information from RGCs to RAs. Similarly to our findings, the authors report spectral responses outside the main visual brain areas (e.g., cerebellum and habenula). However, our results cannot be directly compared due to the different visual fields illuminated in these two works. The presence of neurons with spectral selectivity of response widespread throughout the brain is very interesting. In fact, in teleosts, the role of the OT and other mesencephalic regions as the main centers of integration and processing of sensorial information, including chromatic signals, is known [46], while the possible roles of other anatomical regions in processing visual information are less known. So, we envision neuronal circuit schemes in which the retinal information, relayed to the tectum and here integrated vastly with other visual and non-visual stimuli, is transmitted to the downstream CNS districts for further processing and to elicit responses maintaining, as shown by our data, spectral specificity at the single neuron level. It is known that sensorimotor transformations are produced by complex networks involving different neuron types distributed across multiple brain regions acting to generate a behavior. Naumann et al. [17] describe the neural circuitry underlying the OMR and point out the role of distinct neurons organized in the rostro-caudal direction to allow propagation of the landscape motion information and consequent generation of a motor response. Similarly, our data indicate the presence of neurons whose response is triggered in a spectrally specific manner throughout the larva CNS, thus indicating the presence of “color”-selective circuits with such extension. Thus, we can state that our maps possibly identify neurons which maintain a well-defined spectral identity and are on the pathway for generating “color”-dependent behaviors. In this regard, it was particularly striking to find spectrally selective responses in some spinal cord neurons. Examining the spinal cord at 5 dpf, we observe a relevant number of neurons responding to L_1_, L_2_, and L_3_ radiation but not to L_4_ (Fig. 4c, f, i, l). A reasonable explanation could be associated with the visually guided behaviors elicited by different light wavelengths in zebrafish larvae. It is known that long wavelengths contribute strongly to the OMR, while short wavelengths elicit phototaxis [9]. Indeed, recent works have reported a strong UV avoidance both in larvae and adults, using phototaxis assays on larvae [10] and split tank assays in adults [47]. These behaviors can be most likely explained by the danger represented by strong UV light, particularly in the case of larvae, due to their transparency. In fact, we presume that the stimulation of UV cones will lead to the aforementioned avoidance reflexes, thus triggering a motor response visible in our experiments as activation of spinal cord neurons. Another interesting observation from our maps derives from epiphysis. The zebrafish pineal gland is characterized by photoreceptors with different types of opsins and projection neurons [48–50], and it plays an important role in the regulation of the circadian rhythms. In this work, we observe some epiphysis neurons with strong negative values of *T*, especially for UV radiation, thus highlighting, at the cellular level, the inhibitory effect of short-wavelength light on this organ. Different studies performed in other teleosts and other classes suggested that the pineal projection neurons show a direct sensitivity to light with an inhibitory response in the violet-UV range, and an excitatory response at medium-long wavelengths [51–53]. A recent study carried out on zebrafish [50] has highlighted the presence of a subpopulation of pineal projection neurons exhibiting an excitatory response to blue and green light whose activity is not modulated by RGCs. The inhibitory effect we observed in this work could be associated to a subset of neurons responding in an inhibitory manner to UV light. The comparison maps at different stages of development show a shift in the predominant response from red (at 3 dpf) to UV radiation (at 5 dpf), although there is a generalized lower density of responsive neurons in 3-dpf larvae. However, a direct comparison between 3- and 5-dpf larvae is not possible since at 3 dpf, the larval visual system is still developing and the retina becomes fully functional by 72 hpf [41]. Moreover, important synaptic connections associated with the integration of color and spatial information start developing after 3 dpf, when an improved acuity in zebrafish larvae has been demonstrated [13, 41, 45]. In fact, the first visually evoked responses such as escape behaviors appear between 68 and 72 hpf, while the first OKR between 72 and 80 hpf [11]. This is in accordance with the results obtained in 3-dpf larvae, where we found a negligible level of integration between the different wavelengths (in contrast to 5 dpf data). Furthermore, at 3 dpf, we did not detect any neuron with spectral specificity in the spinal cord, indicating that, at this early stage of development, the circuitries in charge of carrying spectral information down to the spinal cord and, thus, possibly eliciting color-selective motor behaviors, are not yet functional.

## Conclusion

Color vision is a crucial aspect of world perception for most animals. Our measurements provide spectral and anatomical maps of neurons involved in color-driven responses in the zebrafish larva CNS. Besides the well-expected high density of responsive neurons in the visual areas of the mesencephalon (i.e., OT and other RAs, the brain regions specifically involved in processing visual information and integrating it with other sensorial inputs), we found spectrally defined responses also in all other areas down to the spinal cord. Thus, we evidenced neural pathways maintaining spectral information throughout the CNS. These results open the way for a detailed dissection of the circuits responsible for different color-dependent behaviors with ablation and optogenetics experiments. The zebrafish larva is a very simple model yet representative of vertebrate CNS organization, so we expect the paradigm of spectral information propagating through non-visual areas to be relevant in all vertebrates.

## Methods

### Zebrafish maintenance

Adult zebrafish were maintained for breeding at 28 °C on a 14/10-h light/dark cycle according to the standard procedures [54]. Embryos and larvae were raised at 28 °C up to 5 days post-fertilization (dpf) in 40 mL fish water (150 mg/L Instant Ocean, 6.9 mg/L NaH_2_PO_4_, 12.5 mg/L Na_2_HPO_4_, and 1 mg/L methylene blue, pH 7.2) in 9-cm-diameter Petri dish.

All experiments were carried in accordance with the European and Italian law on animal experimentation (D.L. 4 March 2014, n.26), under authorization n. 407/2015-PR from the Italian Ministry of Health.

### Zebrafish larvae preparation

We employed 3- and 5-dpf Tg (elavl3:H2B-GCaMP6s) zebrafish larvae (ZFIN line *fi2Tg*, ref. [55]) with pan-neuronal expression of the nuclear-localized calcium indicator GCaMP6s [56, 57]. The transgene for GCaMP6s expression was carried in a heterozygote albino (*slc45a2*^*b4*^) background. Incross of adults allowed selection of larvae positive for GCaMP and homozygote albino for use in the measurements, so to completely avoid the presence of skin pigments. The mounting procedure has already been described [58]. Briefly, each larva was transferred into a reaction tube (Eppendorf) containing low gelling temperature agarose (A9414, Sigma; 1.5% w/v in fish water) at 38 °C and then quickly drawn into a glass capillary tube (I.D. 0.86 mm, B150-86-10, Sutter Instrument) using a pipette. After gel polymerization, the larva was extruded from the capillary and oriented on the microscope slide by appropriate rotation of the extruded agarose cylinder; orientation was dorsal side up (toward the microscope objective, see Fig. 1b). The cylinder was then covered with a small drop of melted agarose. To minimize movement artifacts, the larva was paralyzed with 4 mM d-tubocurarine (T2379, Sigma) dissolved in fish water (10 min pre-incubation before measurements, followed by washing with fish water). Experiments were performed at room temperature.

### Two-photon calcium imaging and visual stimulation

Imaging during visual stimulation was performed with a two-photon microscope (Thorlabs Bergamo) equipped with a Ti-Sa laser (Coherent Chameleon) tuned to 920 nm and a × 20/0.95 NA water immersion Olympus objective. GCaMP fluorescence was detected using PMT with two band-pass filters (Semrock FF01-520/35) in series. Imaging was performed with a field of view (FOV) of 920 × 720 μm^2^ and a resolution of 0.9 μm/px. A mosaic acquisition was implemented to cover the whole larval encephalon and about one quarter of the spinal cord: this required acquisition of two adjacent FOVs, shifted by about 700 μm along the larva longitudinal axis. To obtain a full 3D acquisition of the CNS volume of interest, *z*-scans were performed on each of the two FOVs. The range of the *z*-scan, sampled with 5 μm steps, was adjusted based on the depth of the CNS area to be imaged (270 μm for the encephalon and about 120 μm for the spinal cord). The total scanned area was 1500 × 720 μm^2^ and variable depth as specified above. Stacks from different samples were registered onto a reference brain [20] (see the “Image registration” section). For visual stimulation, we used four LEDs (Thorlabs M365L2, M420L3, M470L3, and M590L3) with emission spectra centered on the absorption peaks of zebrafish cones. The LEDs are indicated as L_1,_ L_2,_ L_3_, and L_4_ in Fig. 1b. L_2,_ L_3_, and L_4_ were equipped respectively with band-pass filters Semrock FF01-420/10, FF01-469/35, and FF01-600/14 to restrict the relatively broad spectrum of LED emission to a narrow band for selective cone excitation (corresponding to the absorption peaks at 362, 415, 480, and 570 nm of UV, S, M, and L cones, respectively [4, 10, 21, 22]). The LED sources were mounted on the stage of the microscope and coupled into one optical path with three dichroic mirrors (Semrock FF376-Di01, Thorlabs DMLP425R, and DMLP567R, respectively, DM_1_, DM_2_, and DM_3_ in Fig. 1b) so that all stimuli were presented to the larva from the same direction (i.e., dorso-frontally). The intensity of the four LEDs was measured and adjusted at 1 mW/cm^2^. LED flashes (100 ms duration) were presented to the larva in a sequence of 12 regularly paced stimuli (12.5 s apart from each other, see Fig. 1d) and synchronized with the microscope acquisition program (ThorImage) using a NI USB-6002 (National Instrument) controlled by a software written in LabView. Based on the dynamics of the GCaMP6s indicator, we observed that the response of activated neurons typically consisted of a fluorescence spike fully returning to baseline levels in about 12 s from the stimulus (see, for example, Fig. 1f–k). Therefore, in our protocol, we spaced visual stimuli by 12.5 s from each other. Sampling the four stimuli of interest thus requires 50 s for each repetition. To ensure a reliable regression analysis, we chose the minimum set of three repetitions of the stimulus train (Fig. 1d). We did not turn off the PMT during stimulus presentation (and so, we did not temporally separate visual stimulation from recording during the two-photon scan pattern). Two emission filters in series were placed in the detection pathway resulting in a drastic reduction of stimuli bleed-through. Additional file 9: Fig.S9 shows residual stimulus detection visible especially for the shorter wavelengths. During analysis, the frames acquired during the application of the stimulus were removed. The figure demonstrates the total suppression of any possible light artifact from our analysis. Each scan of the FOV required 420 ms. The complete set of stimuli and fluorescence imaging on each plane required 176 s (i.e., 418 frames), comprising an initial resting time of 20 s. The CNS 3D acquisition shown in this work consisted of about 53 planes for the encephalon and 25 for the spinal cord, so that the whole experimental recording required about 3 h 40 min. Additional file 10: movie M1 shows an example of acquisition with a montage of six planes during the full stimulation protocol.


**Additional file 10**: **movie M1**. Imaging during visual stimulation. GCaMP fluorescence recorded in selected planes of the encephalon of a 5dpf larva during stimulation with colored flashes. The movie shows six planes at different depths (50, 75, 100, 125, 150, 175 μm) sampled for 176 s during visual stimulation. The colored spots (shown in their specific color in the movie) indicate the time at which stimuli were presented. The movie plays at 8.4x speed.

### Automatic neuron segmentation and calcium dynamics analysis

For each plane (sampled for 176 s during visual stimulation), a maximum intensity projection (MIP) was produced to obtain the highest contrast for the nuclear-localized calcium indicator (Fig. 1e, left image and top right inset). Automatic segmentation was performed on the MIP image with a Matlab routine modified from Kawashima et al. [59]. This analysis produces a segmentation image that is an overlay of the MIP with the identified neurons highlighted with individual colors (Fig. 1e, right image and bottom right inset). Consistently with the size of each neuron nucleus at the magnification of our imaging, segmented neurons were represented as a cluster of 13 pixels in a pattern 1,3,5,3,1. The fluorescence intensity integrated into the 13-pixel cluster attributed to each cell (*F*(*t*), Fig. 1f) was processed through the 176 s of recording for each plane to obtain the Δ*F*/*F*_0_ normalized traces, as follows. A running average (window size 21 s, step size 0.84 s) was calculated on the *F*(*t*) trace using Matlab *msbackadj* to obtain a baseline (shown in red in Fig. 1f) that was used as a measurement of *F*_0_ in the calculation of Δ*F*/*F*_0_ = (*F*(*t*) − *F*_0_)/*F*_0_.

Each Δ*F*/*F*_0_ trace was then smoothed by applying Matlab *smooth* function (window size 2.1 s). The resulting trace is shown in Fig. 1g.

### Responsive neurons identification

The identification of responsive neurons was based on linear regression analysis on individual neuron Δ*F*/*F*_0_ recordings. The analysis was performed using a Matlab script based, with modifications, on the method described by Miri et al. [60] to measure the degree of responsiveness of each neuron to each spectral stimulus. The time-dependent sequence of the controlled stimuli (Fig. 1d) was convolved with the calcium impulse response function, modeled in our system with an exponential rise time 1.5 s and decay time 2.1 s; these values were found empirically to best match the typical time course of the observed GCaMP6s fluorescence peaks associated with the visual stimuli. The time separation between adjacent stimuli ensured the orthogonality of the four vectors describing the time trajectory of each colored stimulus. These vectors are the columns of the matrix G subsequently used for regression analysis: a four-element vector *β* was obtained by linear regression $\hat{\beta}={G}^T\left(\Delta  F/{F}_0\right)$ with each element representing the regression coefficient for each wavelength in the neuron under analysis. From these coefficients, a *T*-score was calculated as follows:

$$ {T}_i=\frac{\beta_i}{\sqrt{\frac{\varepsilon^2}{n-3}}} $$

With $\hat{\varepsilon}=\left(\Delta  F/{F}_0\right)-\hat{G}\beta$ (which represents the residuals between fitted and measured data); *β*_*i*_ with *i* = 1, 2, 3, and 4 for L_1_, L_2_, L_3_, and L_4_ stimulus, respectively; and *n* = 418 time samples of the recording.

Figure 1h shows an example of a Δ*F*/*F*_0_ trace with the corresponding *T*-values extracted with this analysis for each color and the resulting fitted trace based on the measured *β* values, demonstrating the effectiveness of the regression analysis. Figure 1i–k demonstrates the excellent correspondence between the measured *T* values and Δ*F*/*F*_0_ features, for example, traces with different responses to one or more stimuli in the examined neurons. To automatically and reliably select the color-responsive neurons, a *T* threshold (*T*_Th_) specific for each stimulus was chosen so that the percentage of false positives (false discovery rate, FDR) would be 1% (Additional file 2: Fig. S2). To select the *T*_Th_ value fulfilling this requirement for each stimulus, we compared the normalized *T* distributions measured for each stimulus in the stimulated experiment with that of a control acquired in the absence of visual stimulation (Fig. 2a, b). Since the regression analysis calculates each *T* for the different stimuli based on the specific timing of the spectrally distinct flashes used in the experiment, we submitted the control recording to the same analysis but producing all possible permutations of the *T* values measured, given that no specific stimulus sequence would be preferable to any other. Thus, the control distribution has been made symmetric in all 4-color dimensions. The presence of a subset of spectrally distinct responsive neurons outside the false-positive neurons derived from the control distribution allows setting a threshold and calculating the corresponding FDR as:

$$ \mathrm{FDR}\left({T}_{\mathrm{th}}\right)={\int}_{T_{\mathrm{th}}}^{+\infty}\frac{P_{\mathrm{CTR}}(T)\  dT}{P_{\mathrm{EXP}}(T)\  dT} $$

We selected *T*_Th_ values for each stimulus so that FDR = 0.01.

In addition to the *T* threshold, we implemented a peak analysis in order to select only those neurons responsive above the *T* threshold whose responses were peaked at each stimulus repetition (i.e., 3 peaks for each spectral stimulus). For this analysis, we used Matlab *findpeaks* with a minimum prominence of 0.3 and applied it to the Δ*F*/*F*_0_ traces renormalized each to its maximum level.

### Barcode for identification of multiple stimuli responses

For the identification of the neurons responsive to multiple stimuli, a “T barcode” has been implemented. In the text, we referred to this parameter as “*Tbar*.” At first, the *T* threshold specific for each of the four stimuli and peak analysis (see paragraph above) were applied to all data. The *Tbar* was then calculated for the selected neurons as follows:

$$ Tbar=8\times {\mathrm{L}}_1+4\times {\mathrm{L}}_2+2\times {\mathrm{L}}_3+1\times {\mathrm{L}}_4 $$

where L_1_, L_2_, L_3_, and L_4_ indicate the responses to the four stimuli, taking a value of 1 if the neuron has a *T* value above the threshold (and three response peaks) for that stimulus and 0 otherwise.

In this way, 15 possible values were produced, indicating the unique response of each cell to one stimulus or to a specific combination of stimuli.

### Image registration

In order to map the anatomical distribution of the responsive neurons across the brain, two-photon calcium imaging data were registered onto a zebrafish reference brain following the procedure described in Randlett et al. [20]. Non-rigid image registration was performed using the software CMTK (Computational Morphometry Toolkit, https://www.nitrc.org/projects/cmtk) and the munger wrapper script for CMTK [61, 62]. The anatomical classifications displayed in Figs. 3, 4, 5, and 6 have been obtained reformatting the *z*-stacks of all neurons selected above each stimulus *T*_Th_ (mapped throughout the brain volume of each sample) by applying the transformation obtained in the registration. We next localized and quantified the responsive neurons across the different brain regions using the atlas defined by Randlett et al. [20].

## Supplementary Information

**Additional file 1 **: **Fig.S1**. Spectral specificity of T analysis. T-distributions resulting from analysis of experimental data using a randomized order of stimuli wavelengths in the regression analysis.

**Additional file 2 **: **Fig.S2**. **Choice of threshold for neuron selection.** Calculation of the value of T threshold ensuring a false discovery rate of 1% for each spectral stimulus.

**Additional file 3 **: **Fig.S3**. Statistical quantification of spectral and anatomical identities in 5 dpf larvae. Data shown in Figs. 3c and 4 (right panels C, F, I, L), reported in histogram form and showing also standard errors.

**Additional file 4 **: **Fig.S4**. Anatomical distributions of inhibitory responses at 3 dpf. Anatomical localization and example ΔF/F_0_ trace of neurons displaying a negative response to light in 3 dpf larva.

**Additional file 5 **: **Fig.S5**. Tbar clusters in responsive neurons. Distributions of neuronal spectral responses classified with Tbar and shown as a function of their overall response to stimuli (quantified by T_4D_). Comparison of emergence of the different Tbar classes for two different choices of T thresholds.

**Additional file 6 **: **Fig.S6**. Neurons spectral identity: standard vs shuffled pattern of visual stimuli. Analysis of the dependence of neuronal spectrally-selective responses on the order of stimuli presentation.

**Additional file 7 **: **Fig.S7**. Calcium response dynamics. Distributions of peak time points for responses measured in experiments with visual stimulation and in unstimulated controls.

**Additional file 8 **: **Fig.S8**. T distributions at different stimulus intensities for stimulated and control larvae at 5 dpf. Comparison of neuronal response distributions for the four different wavelengths at three different powers.

**Additional file 9 **: **Fig.S9**. Suppression of light artefacts. Illustration of elimination of light artefacts due to LED flash bleed-through in PMT detection by frame elimination.

## Data Availability

All data generated or analyzed during this study are included in this article and its supplementary information files (Additional files S1, S2, S3, S4, S5, S6, S7, S8, S9, and Additional movies M1). The datasets used and/or analyzed during the current study are available from the corresponding author on reasonable request.
