# Supplementary material for: Colored visual stimuli evoke spectrally tuned neuronal responses across the central nervous system of zebrafish larvae
Source: BMC Biol. 2020 Nov 27;18:172. doi: 10.1186/s12915-020-00903-3 (PMC7694941; doi:10.1186/s12915-020-00903-3)
Supplement: Supplementary file 2 — Additional file 1 : Fig.S1. Spectral specificity of T analysis. T-distributions resulting from analysis of experimental data using a randomized order of stimuli wavelengths in the regression analysis. [file 12915_2020_903_MOESM1_ESM.docx]

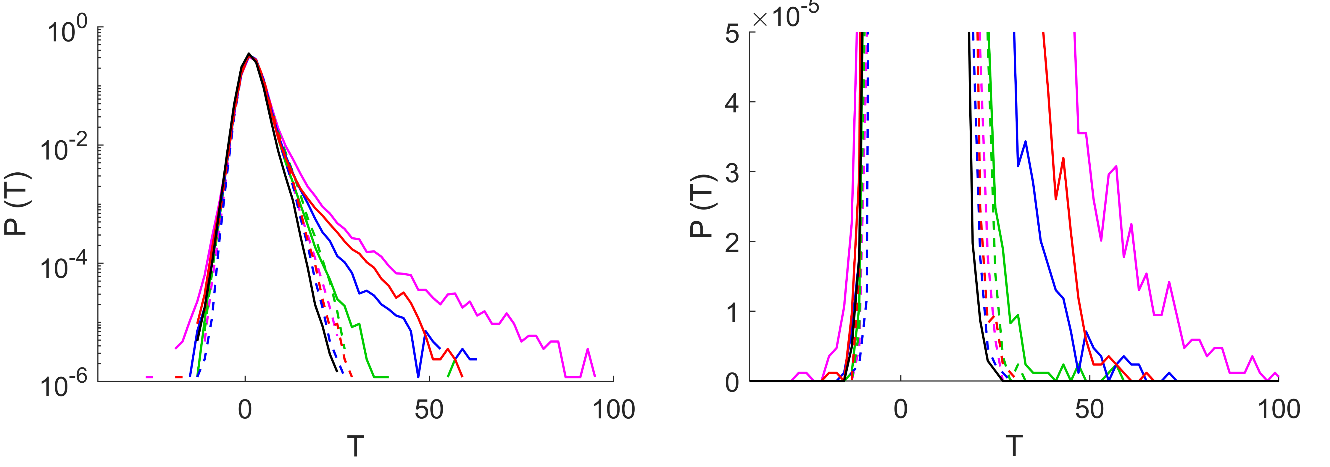

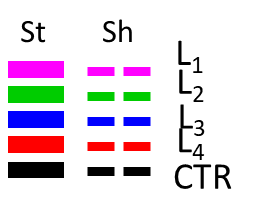


A

B

|  | **Standart** | **Shuffled** | **Percentage** |
| --- | --- | --- | --- |
| **L_1_** | 5048 | 233 | 4.6% |
| **L_2_** | 56 | 7 | 12.5% |
| **L_3_** | 745 | 10 | 1.3% |
| **L_4_** | 2614 | 113 | 4.3% |

**Additional file 1: Figure S1. Spectral specificity of T analysis**. To demonstrate the spectral specificity of T values obtained with regression analysis, we further analyzed our data by applying a shuffled order of regressors (L_2_, L_1_, L_4_, L_3_, L_3_, L_4_, L_1_, L_2_, L_4_, L_3_, L_2_, L_1_), thus permuting the identity of the stimuli compared to the order presented in the experimental measurements. For simplicity, we refer to this pattern as “shuffled”, while we term “standard” the protocol of visual stimulation used in our measurements (Fig. 1D). Panels A-B show normalized T distributions across 7 larvae at 5 dpf comparing experimental data analyzed with standard order of regressors (solid colored traces), with the shuffled pattern of regressors (dotted colored traced), and controls (black traces) data (see legend). T distributions were plotted both in logarithmic scale **(A)** and in linear scale **(B)** to provide a better view in different ranges. The experimental and control distributions analyzed with standard order of regressors are the same data shown in Fig. 2A of the manuscript. The distributions clearly show that the shuffled data fall onto the controls, whereas the data analyzed with the standard protocol identify responsive neurons characterized by higher T values. The table quantifies the number of neurons responding to one of the four stimuli (selected by applying T threshold and peak analysis criteria, see materials and methods) between standard and shuffled analysis. The numbers reported are the neurons above threshold for each stimulus. The drastic reduction of the number of neurons emerging from shuffled analysis (the percentages of these in respect to standard are reported in the table) demonstrates the robustness of analysis and specificity of T with respect to the spectral identity of the stimuli.
