# Supplementary material for: Colored visual stimuli evoke spectrally tuned neuronal responses across the central nervous system of zebrafish larvae
Source: BMC Biol. 2020 Nov 27;18:172. doi: 10.1186/s12915-020-00903-3 (PMC7694941; doi:10.1186/s12915-020-00903-3)
Supplement: Supplementary file 3 — Additional file 2 : Fig.S2. Choice of threshold for neuron selection. Calculation of the value of T threshold ensuring a false discovery rate of 1% for each spectral stimulus. [file 12915_2020_903_MOESM2_ESM.docx]

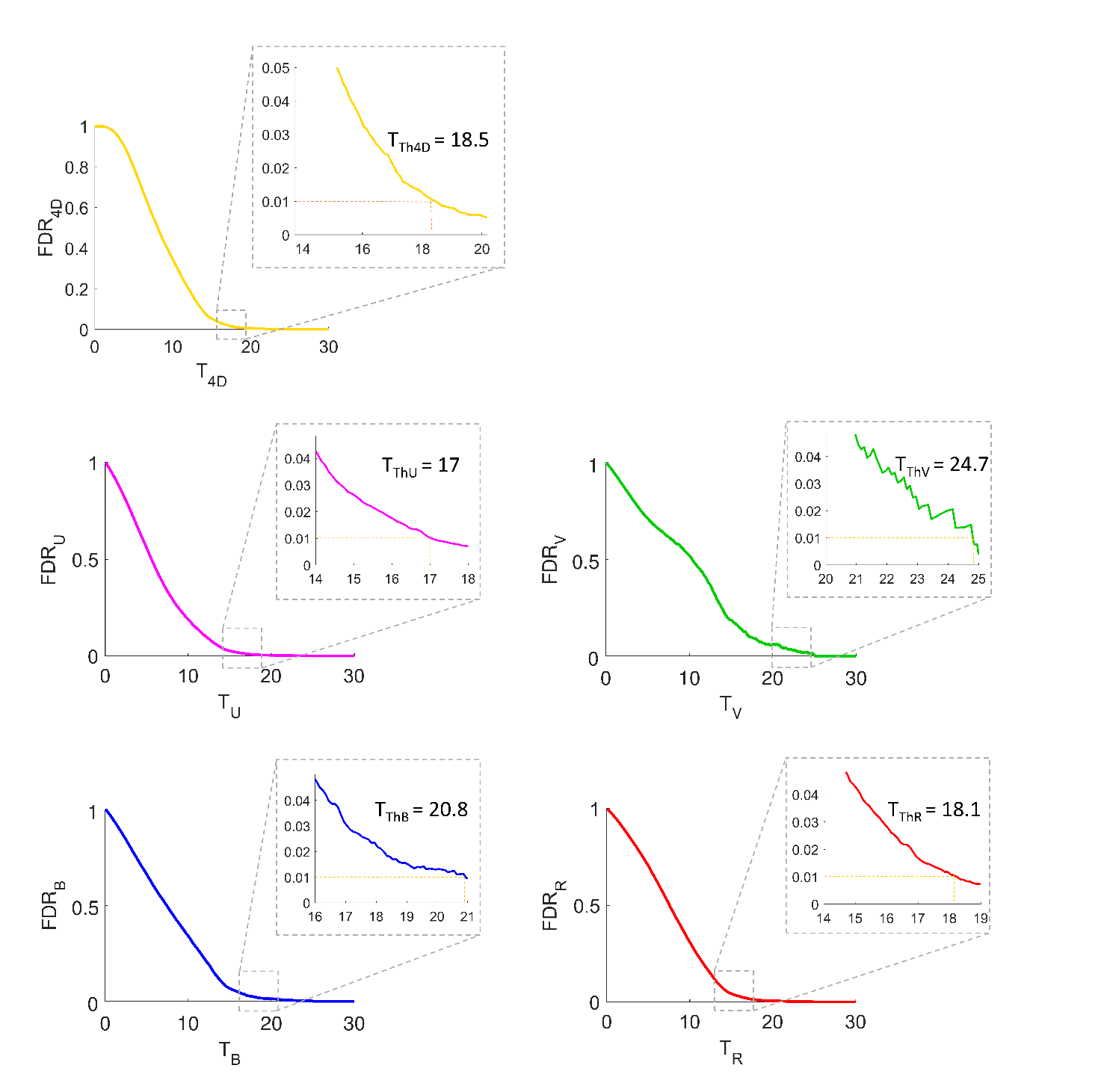


**Additional file 2: Figure S2. Choice of threshold for neuron selection**. Top panel: plot of False Discovery Rate (FDR) used to choose a T_4D_ threshold (T_Th4D_) to automatically select neurons responsive to any of the stimuli we presented in our experiments with a confidence of 99%. The same criterion has been applied to choose T thresholds named T_ThU_, T_ThV_, T_ThB_, T_ThR_, for the selection of L_1_, L_2_, L_3_ and L_4_ responsive neurons, respectively, always ensuring FDR=0.01. T thresholds values are shown in the insets and applied to all subsequent analysis.
