# Supplementary material for: Colored visual stimuli evoke spectrally tuned neuronal responses across the central nervous system of zebrafish larvae
Source: BMC Biol. 2020 Nov 27;18:172. doi: 10.1186/s12915-020-00903-3 (PMC7694941; doi:10.1186/s12915-020-00903-3)
Supplement: Supplementary file 4 — Additional file 3 : Fig.S3. Statistical quantification of spectral and anatomical identities in 5 dpf larvae. Data shown in Figs. 3c and 4 (right panels C, F, I, L), reported in histogram form and showing also standard errors. [file 12915_2020_903_MOESM3_ESM.docx]

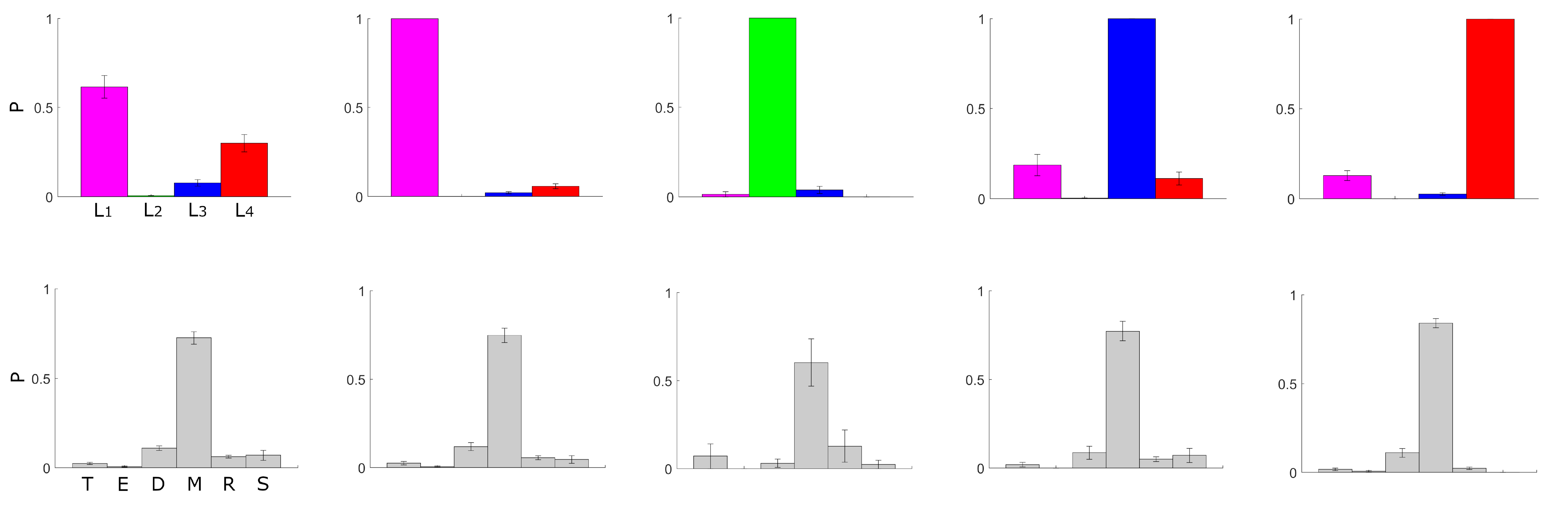


**Additional file 3: Figure S3. Quantification of spectral and anatomical identities in 5 dpf larvae (see Fig. 3-4 in the main manuscript).** From left to right: same data shown in bar form in Fig. 3C and Fig. 4C, F, I, L, respectively. Here we additionally report error bars (stderr, N=7 larvae).
