# Supplementary material for: Colored visual stimuli evoke spectrally tuned neuronal responses across the central nervous system of zebrafish larvae
Source: BMC Biol. 2020 Nov 27;18:172. doi: 10.1186/s12915-020-00903-3 (PMC7694941; doi:10.1186/s12915-020-00903-3)
Supplement: Supplementary file 5 — Additional file 4 : Fig.S4. Anatomical distributions of inhibitory responses at 3 dpf. Anatomical localization and example ΔF/F0 trace of neurons displaying a negative response to light in 3 dpf larva. [file 12915_2020_903_MOESM4_ESM.docx]

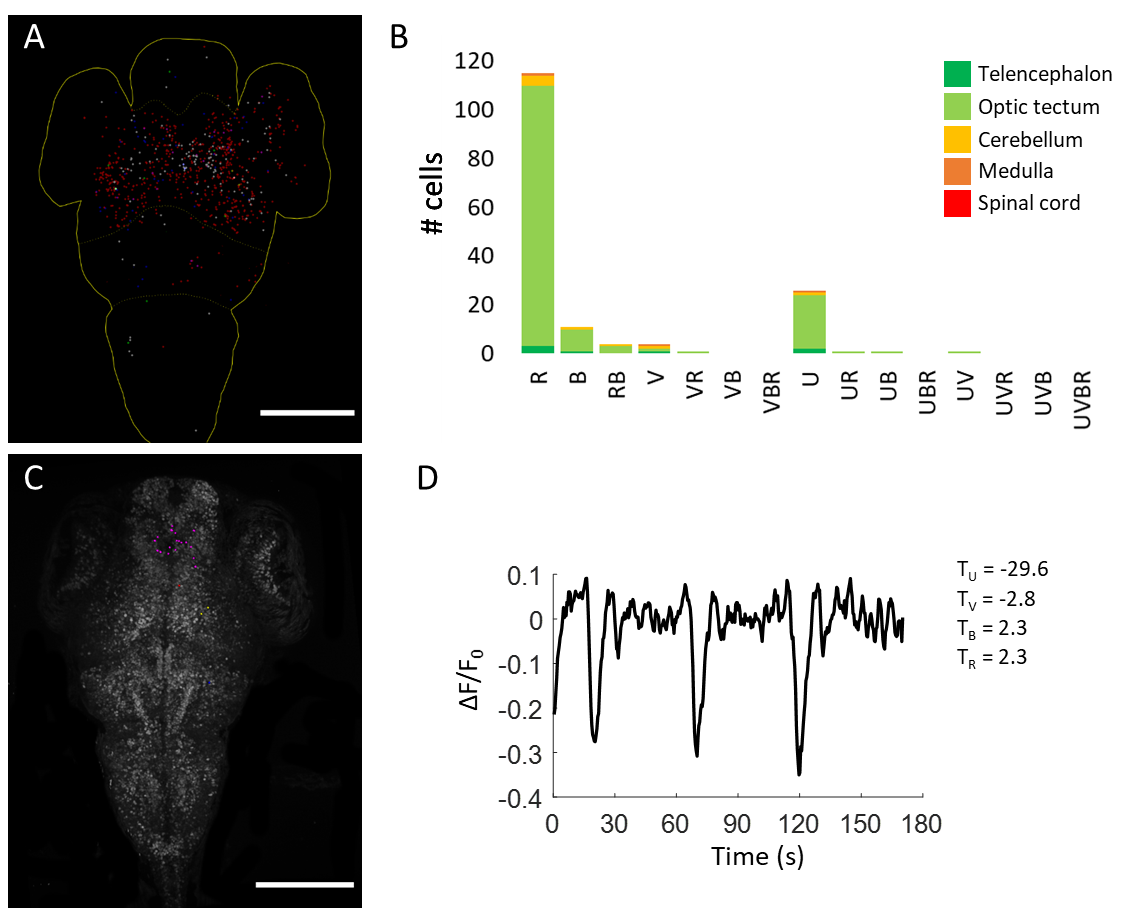


**Additional file 4: Figure S4. Anatomical distribution of inhibitory responses at 3 dpf.** Left panel: T-negative neurons identified specifically for L_1_ stimulus (magenta dots) mapped in larvae at 3 dpf superimposed to a reference brain (light yellow outlines) for anatomical localization. The image highlights the localization of neurons responding in an inhibitory manner to L_1_ radiation in the pineal region. Right panel: ΔF/F_0_ trace of an epiphysis neuron displaying a negative T value for L_1_ stimulus as indicated by the T score values shown in the figure. Scale bar: 150 µm.
