# Supplementary material for: Colored visual stimuli evoke spectrally tuned neuronal responses across the central nervous system of zebrafish larvae
Source: BMC Biol. 2020 Nov 27;18:172. doi: 10.1186/s12915-020-00903-3 (PMC7694941; doi:10.1186/s12915-020-00903-3)
Supplement: Supplementary file 6 — Additional file 5 : Fig.S5. Tbar clusters in responsive neurons. Distributions of neuronal spectral responses classified with Tbar and shown as a function of their overall response to stimuli (quantified by T4D). Comparison of emergence of the different Tbar classes for two different choices of T thresholds. [file 12915_2020_903_MOESM5_ESM.docx]

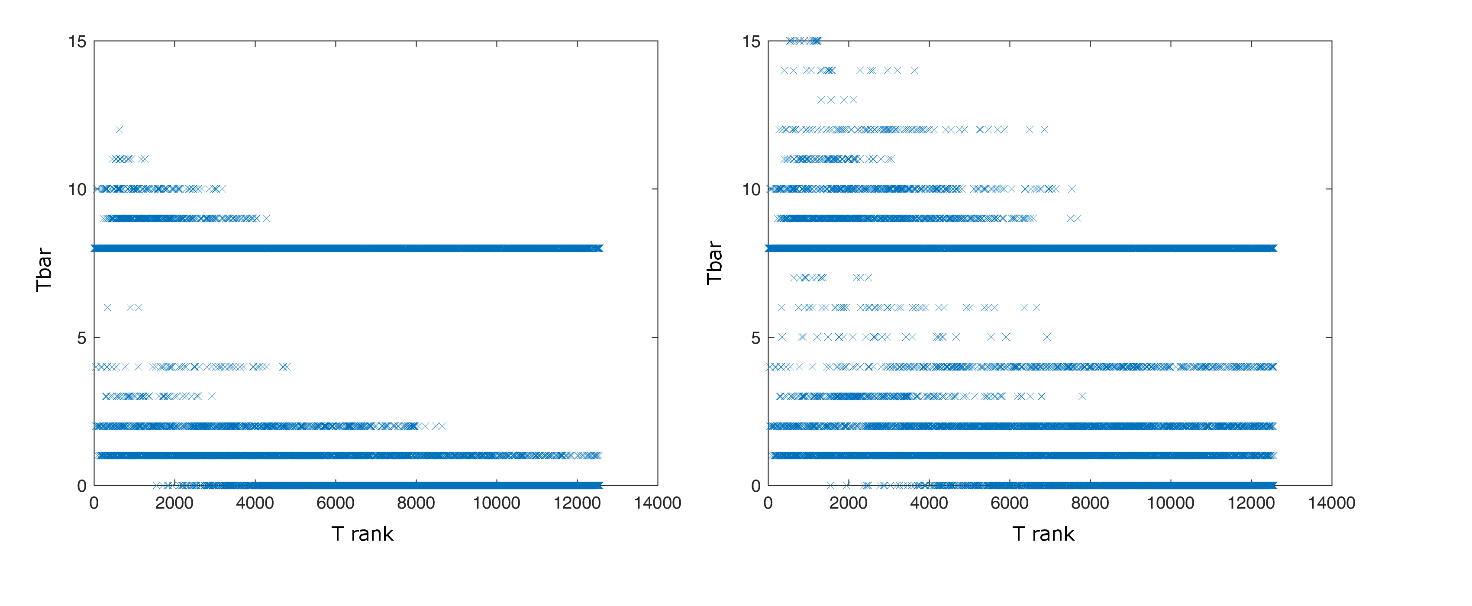


**Additional file 5: Figure S5.** **Tbar clusters in responsive neurons.** Neurons from the seven experimental larvae are ranked based on their T_4D_ value (see main text). Each neuron is plotted as an x on the vertical axis value corresponding to its Tbar class. The left graph shows neurons classified for their Tbar class based on the T thresholds used throughout the paper. The data show a clear and strong emergence of the classes shown in Fig. 5. The graph on the right was plotted simply changing the threshold criterion and setting the threshold to 15 for the four stimuli. This graph shows that the Tbar clusters do not qualitatively change, while, of course, their numerical consistency increases. We should note that this increase of numerical consistency will indeed comprise responsive neurons (i.e. reduce false negatives) but at the expense of an increase of false positives. Thus, the data show that the Tbar clusters do not depend significantly on the choice of threshold.
