# Supplementary material for: Colored visual stimuli evoke spectrally tuned neuronal responses across the central nervous system of zebrafish larvae
Source: BMC Biol. 2020 Nov 27;18:172. doi: 10.1186/s12915-020-00903-3 (PMC7694941; doi:10.1186/s12915-020-00903-3)
Supplement: Supplementary file 7 — Additional file 6 : Fig.S6. Neurons spectral identity: standard vs shuffled pattern of visual stimuli. Analysis of the dependence of neuronal spectrally-selective responses on the order of stimuli presentation. [file 12915_2020_903_MOESM6_ESM.docx]

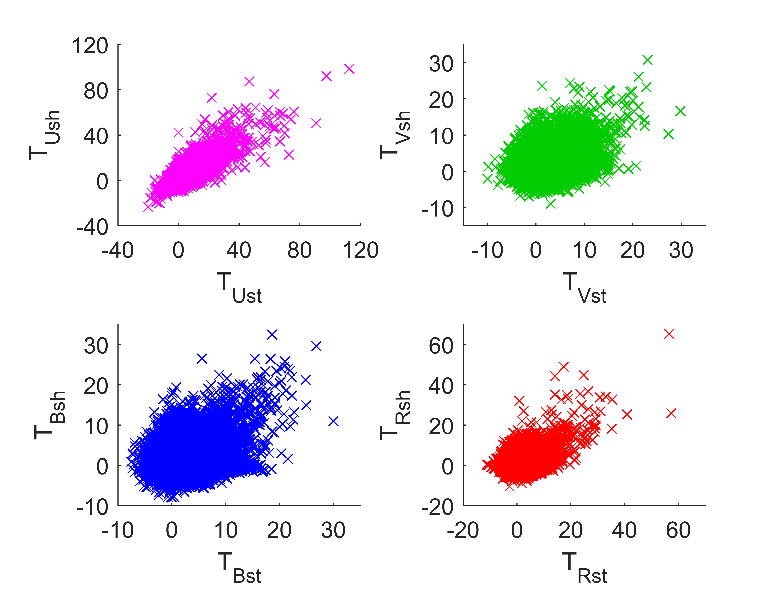


A


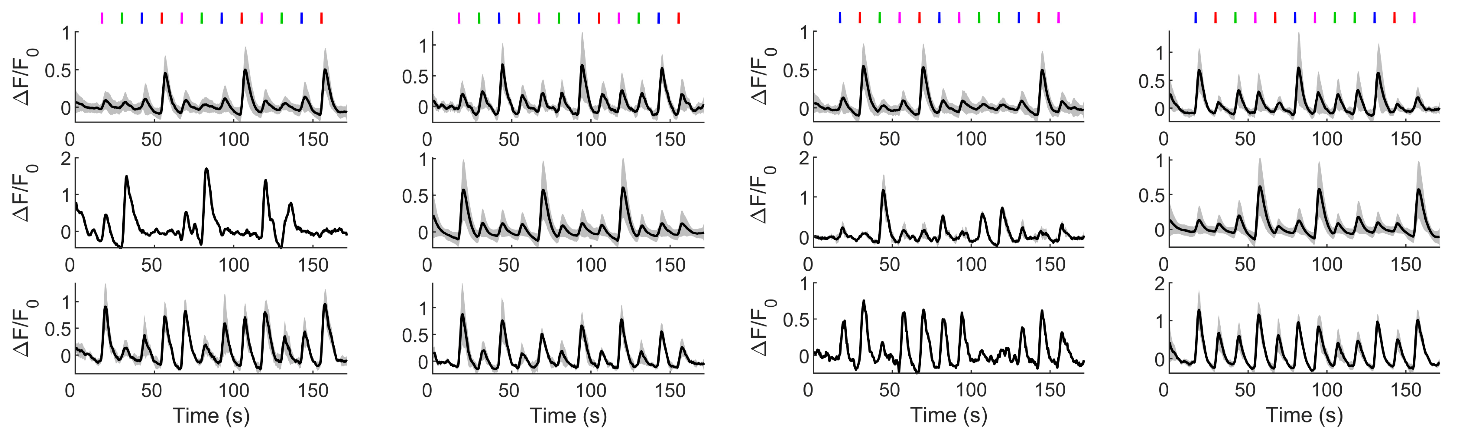


1

2

4

8

10

9

1

2

4

8

10

9

B

**Additional file 6: Figure S6. Neurons spectral identity: standard vs shuffled pattern of visual stimuli.** To further demonstrate neurons specificity of response to one or multiple spectral stimuli, measurements have been performed by applying a stimulation pattern with a shuffled order of the 12 stimuli. We recorded neuronal activity on the same planes with two different stimulus patterns, waiting 120 s between the two consecutive acquisitions. We adopted our standard protocol of stimulation consisting of L_1_, L_2_, L_3_, L_4_ pattern presented in triplicate, and the shuffled pattern L_3_, L_4_, L_2_, L_1_, L_4_, L_3_, L_1_, L_2_, L_2_, L_3_, L_4_, L_1_. For simplicity, we refer to the first as “standard” and to the second as “shuffled”. We recorded activity with both stimulation protocols for 176 s on about 10 planes sampled with 20 µm z-step (N=3 larvae at 5 dpf, total number of ROIs analyzed 45766). In order to investigate if the same neuron maintains a specific spectral response aside from the order of the stimuli, we produced four correlation plots comparing the responses (i.e. the T values) to the four stimuli between standard and shuffled data. We then classified the spectral responses in terms of Tbar (see main manuscript). **(A)** Correlation plots of T values for each of the four stimuli (L_1_-magenta, L_2_-green, L_3_-blue, L_4_-red) between data acquired with the standard (st) and shuffled (sh) protocol of visual stimulation. **(B)** Average ΔF/F_0_ traces (black line: mean; grey area: error shading, standard deviation) of the most representative T bar classes (Tbar values 1, 2, 4, 8, 9, 10, indicated with the number in each panel, which mean responses to L_4_ only, L_3_ only, L_2_ only, L_1_ only, L_1_&L_4_, L_1_&L_3_, respectively), calculated for both stimulation protocols (standard, left six panels; shuffled, right six panels). Stimulus time points are indicated in their respective order and color.
