# Supplementary material for: Colored visual stimuli evoke spectrally tuned neuronal responses across the central nervous system of zebrafish larvae
Source: BMC Biol. 2020 Nov 27;18:172. doi: 10.1186/s12915-020-00903-3 (PMC7694941; doi:10.1186/s12915-020-00903-3)
Supplement: Supplementary file 8 — Additional file 7 : Fig.S7. Calcium response dynamics. Distributions of peak time points for responses measured in experiments with visual stimulation and in unstimulated controls. [file 12915_2020_903_MOESM7_ESM.docx]

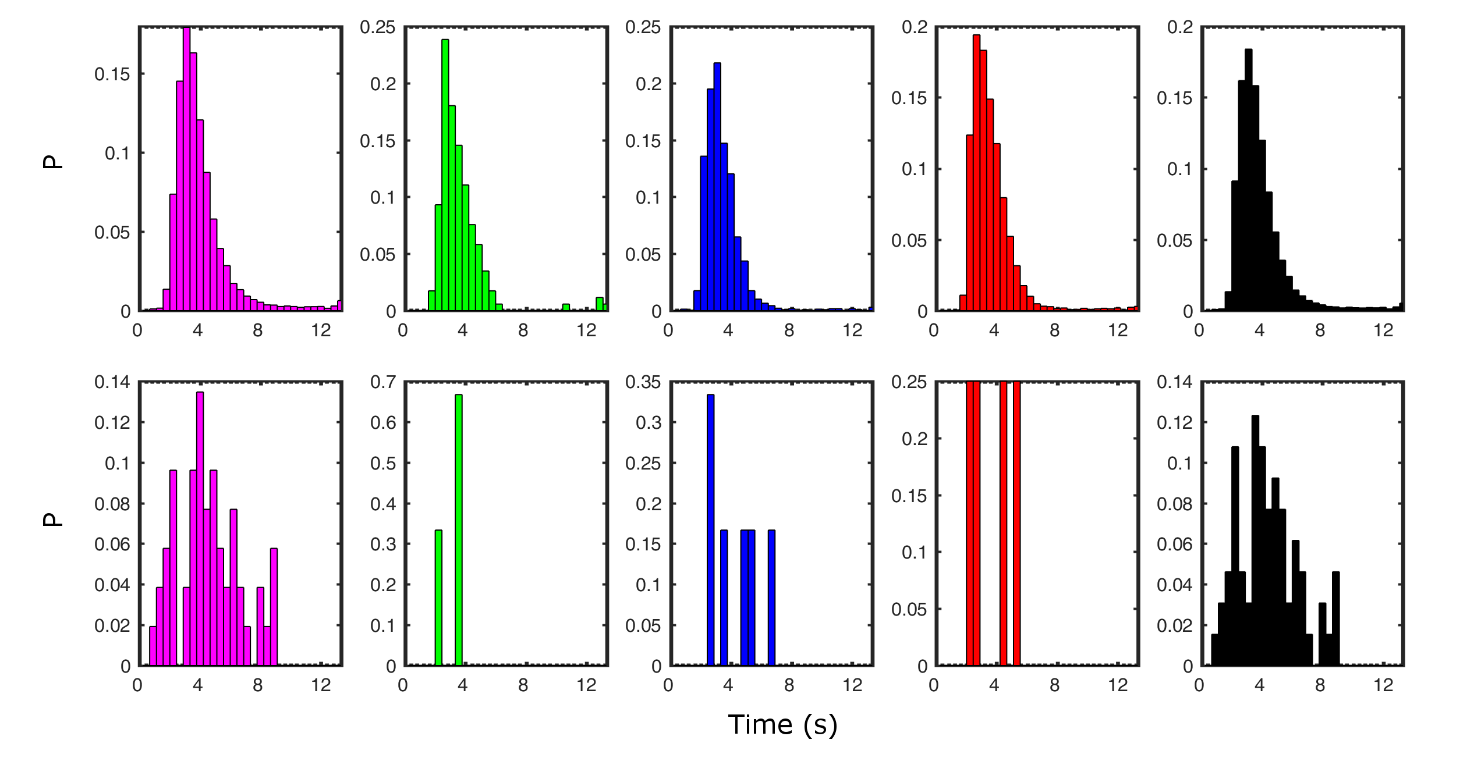


**Additional file 7: Figure S7. Calcium response dynamics.** The graphs show histograms of the distributions of peak times for the neurons responsive to each spectral stimulus (i.e. selected based on the respective thresholds and shown with its color code in the first four panels) and the cumulative distribution of all responsive neurons regardless of the stimulus wavelength (rightmost panels in each row, black bars). The top graphs show data measured on the experimental larvae (N=7) that were visually stimulated, the bottom graphs show data measured on control larvae (N=5) that were not visually stimulated. The time shown on the x-axis is measured relative to the presentation of the stimulus (t=0).
