# Supplementary material for: Colored visual stimuli evoke spectrally tuned neuronal responses across the central nervous system of zebrafish larvae
Source: BMC Biol. 2020 Nov 27;18:172. doi: 10.1186/s12915-020-00903-3 (PMC7694941; doi:10.1186/s12915-020-00903-3)
Supplement: Supplementary file 9 — Additional file 8 : Fig.S8. T distributions at different stimulus intensities for stimulated and control larvae at 5 dpf. Comparison of neuronal response distributions for the four different wavelengths at three different powers. [file 12915_2020_903_MOESM8_ESM.docx]

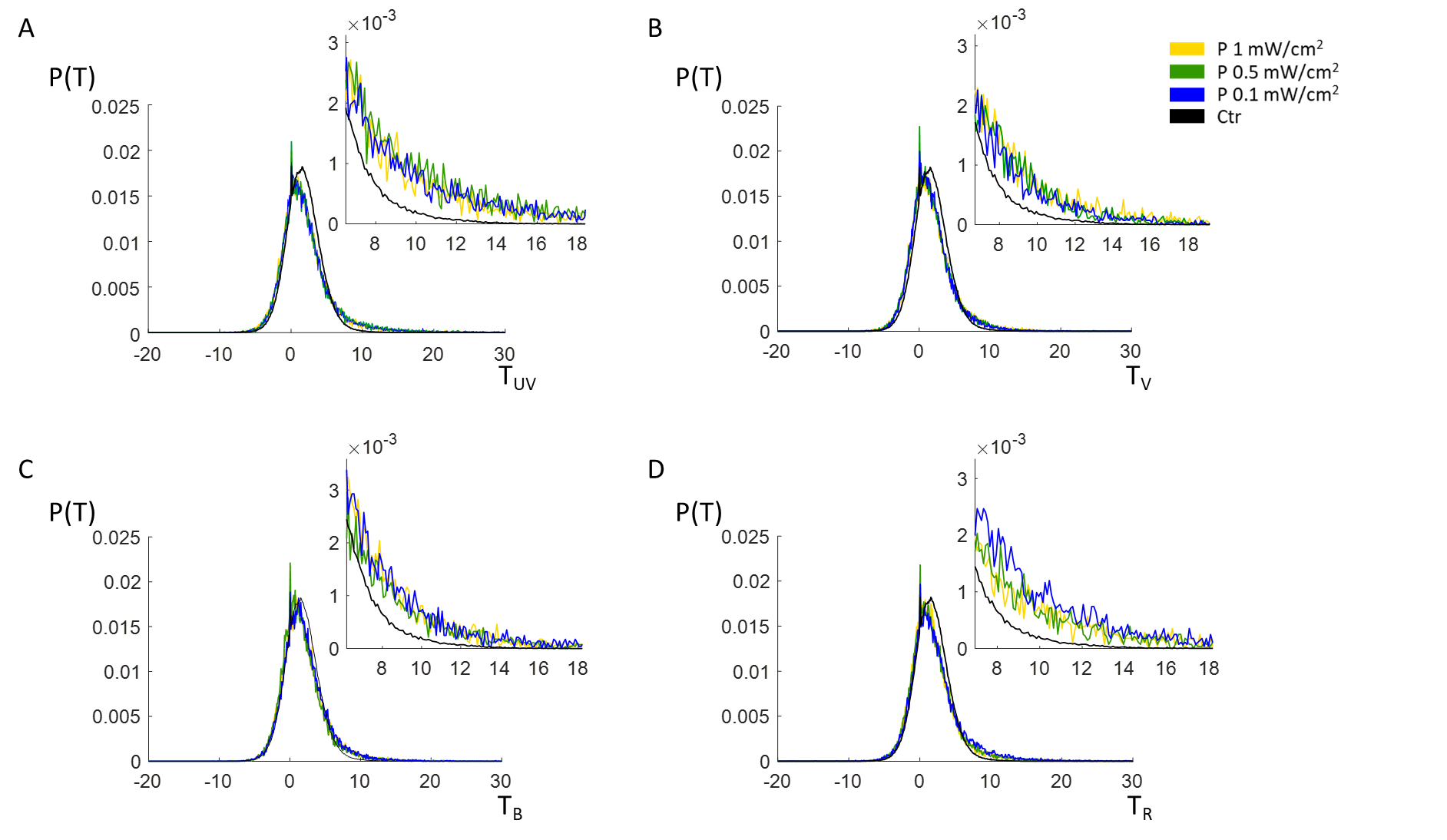


**Additional file 8: Figure S8. T distributions at different stimulus intensities for stimulated and control larvae at 5 dpf.** Normalized T distributions obtained at three different stimulus intensities (1 mW/cm^2^, 0.5 mW/cm^2^, 0.1 mW/cm^2^, yellow, green and blue traces, respectively; see legend) compared with control (black trace). T distributions are shown in linear scale and are reported for each stimulus (**A**, L_1_; **B**, L_2_; **C**, L_3_; **D**, L_4_). The insets on the left are enlarged views of the graphs.
