# Supplementary material for: Colored visual stimuli evoke spectrally tuned neuronal responses across the central nervous system of zebrafish larvae
Source: BMC Biol. 2020 Nov 27;18:172. doi: 10.1186/s12915-020-00903-3 (PMC7694941; doi:10.1186/s12915-020-00903-3)
Supplement: Supplementary file 10 — Additional file 9 : Fig.S9. Suppression of light artefacts. Illustration of elimination of light artefacts due to LED flash bleed-through in PMT detection by frame elimination. [file 12915_2020_903_MOESM9_ESM.docx]

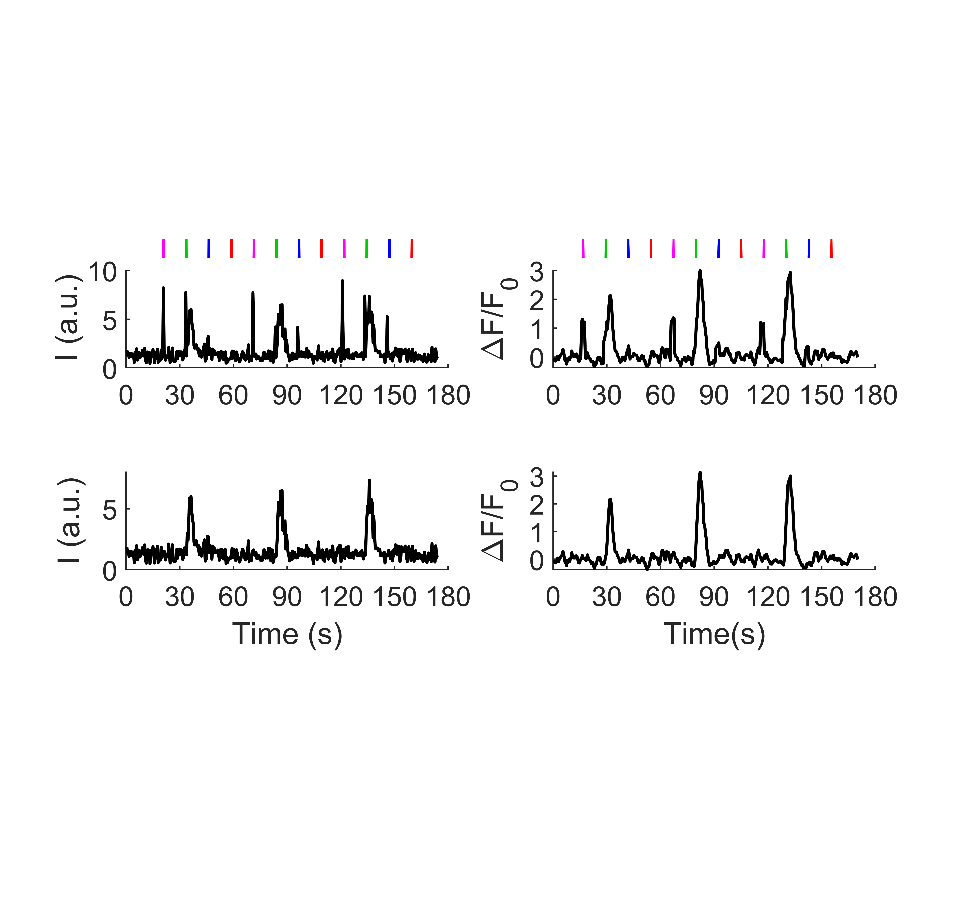


A

B

**Additional file 9: Figure S9. Suppression of light artefacts.** **(A)** GCaMP time fluorescence raw intensity data measured from PMT in ROI selected for the example without removal of the frames where the stimuli appear (top panel). Stimulus time points are indicated above the trace. The L_1_, L_2_ and L_3_ light flashes are clearly visible as signal spikes in the trace (with decreasing amplitude for increasing wavelengths from L_1_ to L_3_), due to bleed-through of the LED light in the detection pathway; the L_4_ stimulus (red) is not detected, being entirely filtered out by the fluorescence filters placed in front of the PMT. Bottom panel shows the same trace after elimination of the frames with the stimuli. **(B)** Corresponding $\Delta F/F_{0}$ traces obtained by applying Matlab *msbackadj* and *smooth method* (see material and methods). The top trace is shown only to display what the effect would be on the $\Delta F/F_{0}$ traces if stimulus frames were not removed. The bottom trace is an example of the type of data analyzed throughout our work. The delayed and fairly slow onset of GCaMP6s response insures that the elimination of the frames with the stimuli does not affect at all the detection of neuronal response.
